# Skeletal Muscle Tissue Engineering: From Tissue Regeneration to Biorobotics

**DOI:** 10.34133/cbsystems.0279

**Published:** 2025-05-15

**Authors:** Maira Z. Cordelle, Sarah J. B. Snelling, Pierre-Alexis Mouthuy

**Affiliations:** Botnar Research Centre, Nuffield Department of Orthopaedics, Rheumatology, and Musculoskeletal Sciences, University of Oxford, Oxford OX3 7LD, UK.

## Abstract

With its remarkable adaptability, energy efficiency, and mechanical compliance, skeletal muscle is a powerful source of inspiration for innovations in engineering and robotics. Originally driven by the clinical need to address large irreparable muscle defects, skeletal muscle tissue engineering (SMTE) has evolved into a versatile strategy reaching beyond medical applications into the field of biorobotics. This review highlights recent advancements in SMTE, including innovations in scaffold design, cell sourcing, usage of external physicochemical cues, and bioreactor technologies. Furthermore, this article explores the emerging synergies between SMTE and robotics, focusing on the use of robotic systems to enhance bioreactor performance and the development of biohybrid devices integrating engineered muscle tissue. These interdisciplinary approaches aim to improve functional recovery outcomes while inspiring novel biohybrid technologies at the intersection of engineering and regenerative medicine.

## Introduction

Skeletal muscle is a highly differentiated and organized tissue that makes up over 40% of human body weight [1–3]. It converts chemical energy into mechanical energy to generate force, maintain posture, and produce movement [4]. As an actuation system, skeletal muscle possesses unique characteristics, such as mechanical compliance, energy efficiency, fine motor control, and adaptability through training, allowing it to perform a wide range of tasks requiring varying levels of precision and strength [5]. Remarkably, skeletal muscle has a strong ability to regenerate. Thus, for most muscle injuries, rest and rehabilitation are enough to allow muscle tissue to repair itself naturally. Despite this remarkable regeneration capacity, substantial volumes of muscle loss do not naturally recover and require interventional support. When volumetric muscle loss (VML) surpasses 15% to 20%, patients currently have no alternative but to undergo an autologous muscle flap graft [3,6]. Although this procedure is the gold standard for the treatment of VML, it is associated with major drawbacks including donor site availability and morbidity, extensive surgery time, limited functionality, and risk of graft failure [7] Additional details about VML can be found in Box 1.

Box 1.  VML epidemiology and clinical need

The incidence of musculoskeletal pathologies is on the rise, driven by the expanding aging population [8]. In the last 30 years, the burden of musculoskeletal disorders has surged by over 30%. These conditions substantially impair movement and coordination, leading to premature exit from the workforce, diminished overall health, and limited engagement in community life. Affecting roughly 1.71 billion individuals, they represent the leading cause of disability worldwide [9]. Musculoskeletal disorders encompass more than 150 different conditions, affecting bones, joints, ligaments, tendons, and muscles [10]. VML injuries are a widespread category of muscle afflictions, associated with an extensive loss of function. Indeed, a loss of muscle weight as small as 10% to 20% can cause a 30% to 90% loss of muscle overall strength [11]. VML can have various causes including crush injuries, penetrative trauma and blast, burns, surgical resection of aggressive malignant tumor, secondary trauma-like compartment syndrome, and comorbidity to open bone fracture [7]. Affecting all age categories, VML causes permanent loss of muscle strength and range of motion, associated with long-term inflammation and extensive fibrosis [12]. These manifestations of VML injuries can lead to chronic disability and overall lower physical activity levels, which then contribute to a general health decline in patients. As there are currently no satisfactory treatments available for VML, there is a clinical need for the development of novel therapies that will allow for a full restoration of muscle function following VML injuries.

The development of engineered muscle grafts presents a promising alternative strategy to tackle those shortcomings [3,6]. Producing such muscle tissue constructs is the main focus of the field of skeletal muscle tissue engineering (SMTE). The general SMTE approach consists of utilizing bioreactor systems to generate tissue in vitro using scaffolds, human or animal cells, and appropriate growth conditions (i.e., chemical environment and relevant external stimuli) [13,14]. While the intricate interplay between these various parameters makes the production of healthy and functional skeletal muscle tissue in vitro an ongoing challenge, current SMTE approaches provide exciting opportunities for medical applications. Beyond regenerative medicine, engineered muscle constructs also hold considerable potential in biorobotics. This emerging interdisciplinary field merges principles from robotics, biology, and engineering to develop robotic systems that either take inspiration from biological structures or directly incorporate living components. While “biorobotics” has been defined in various ways, including as “the science and engineering of applying robotics to problems regarding biology and medicine” [15], the definition that best aligns with the scope of this review emphasizes the integration of biological materials, systems, and principles into robotic technology [16]. Within the context of SMTE, biorobotics encompasses both bioinspired robots that replicate muscle-like actuation and biohybrid robots that integrate engineered skeletal muscle tissue into artificial frameworks, creating adaptive systems with enhanced functionality and responsiveness [5]. Successfully integrating engineered muscle into robotic systems requires careful optimization of all core components of SMTE to achieve the desired properties for specific applications. Among these components, scaffold design is particularly critical, as the mechanical properties, porosity, and biodegradability of the scaffold directly influence the structural integrity and contractile performance of the engineered muscle tissue. The selection of the cell type is also a key consideration to achieve cost-effective long-term scale-up of production while maintaining ethical standards. Additionally, bioreactor design plays a fundamental role throughout the lifespan of integrated muscle constructs. In the early stages, bioreactors provide a controlled environment that supports the development of functional, contractile muscle tissue. Bioreactor chambers, which effectively host tissue constructs, should ensure their long-term sterility and sustained functionality.

This intersection between SMTE and robotics not only advances regenerative medicine but also opens new possibilities for both medical and technological applications. In this context, this review aims to discuss the current trends in SMTE. After outlining the hierarchical structure of skeletal muscle and its natural regeneration pathways, the strategies developed for engineering skeletal muscle will be reviewed, with an emphasis on 3 key domains: scaffold production, cell types, and the role of external stimuli in tissue maturation. Particular attention will be given to the bioreactor systems that support the growth and development of engineered muscle. Additionally, this review will explore the complementary relationship between SMTE and robotics, highlighting the promising contribution of advanced robotic platforms, such as humanoid robots, in providing relevant stimulations to engineered muscle constructs, as well as the current advances in the development of engineered skeletal muscle-actuated biohybrid robots.

## Skeletal Muscle Structure and Function

**Fig. 1.**
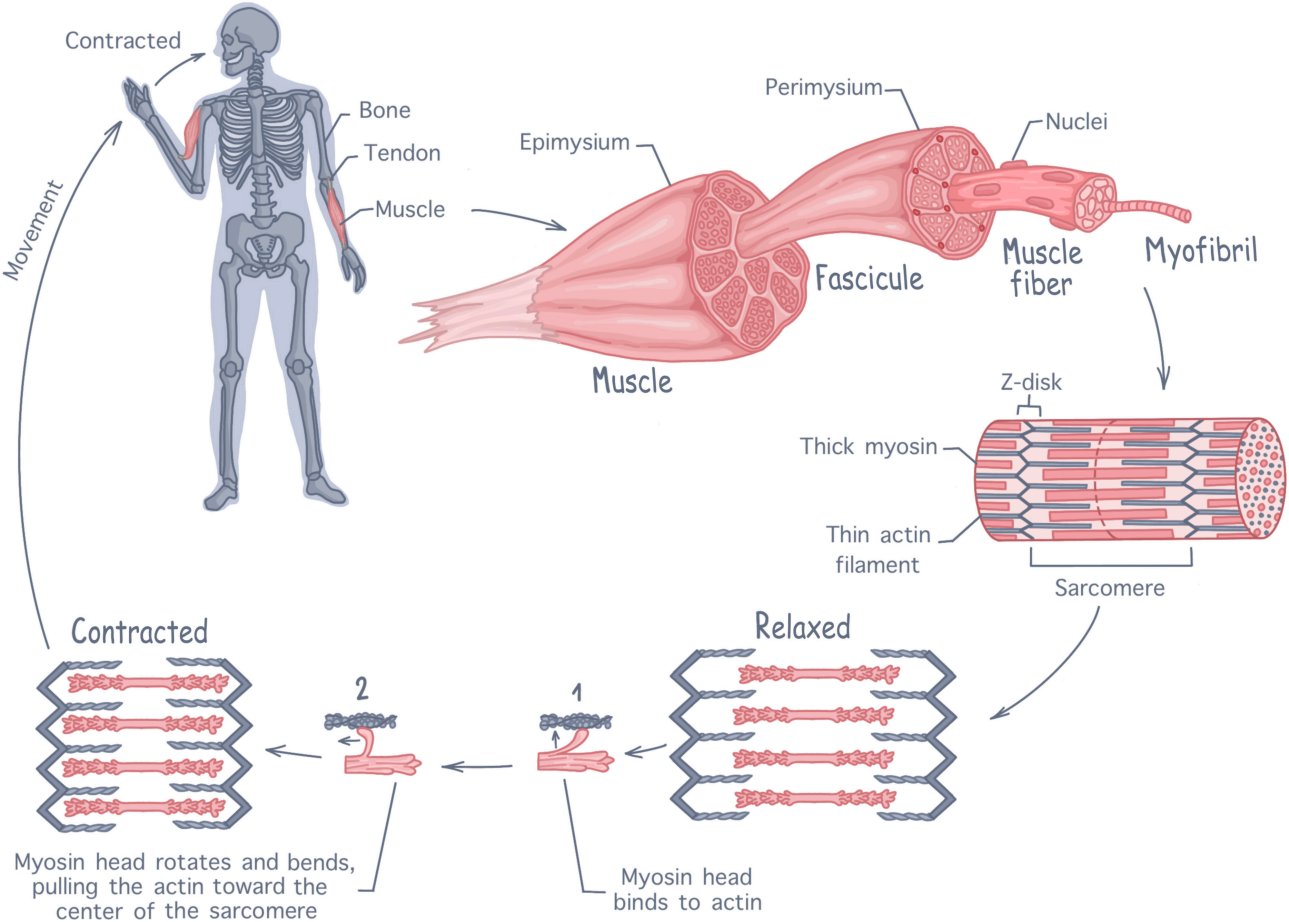
Anatomy of skeletal muscle tissue, illustrating the hierarchical structure from whole muscle down to sarcomeres. The contraction process involves interactions between actin and myosin filaments within the sarcomeres to generate force.

Skeletal muscle is a complex tissue, with a highly organized 3-dimensional (3D) structure intimately linked to its main function: generating force to produce movement [2]. There are over 400 distinct skeletal muscles in the human body, attached to the skeleton by tendons to transmit the generated forces into motion. As shown in Fig. 1, each individual muscle is encapsulated by the epimysium, a dense connective tissue (made of coarse collagen fibers) that protects the organ and passively participate in force transmission [17]. The muscle in itself is composed of muscle fiber (myofibers) bundles, called fascicles, that are each surrounded by another layer of connective tissue, the perimysium. Each myofiber is a highly specialized, elongated, and multinucleated muscle cell, formed by the fusion of undifferentiated muscle cells (myoblasts). Each fiber contains hundreds of nuclei, located at the periphery of the cells, and thousands of long protein filaments arranged in parallel forming rod-like organelles called myofibrils. Myofibrils are the contractile machinery of the myofibers and can make up to 75% of a muscle’s total volume. They are made up of repeating fundamental units called sarcomeres. Each sarcomere consists of an alternating arrangement of thick myosin filaments overlapping with thin actin filaments, which are laterally delimitated by plate-like structure called Z-disks [18]. At the molecular level, sarcomeric contraction is a movement of the myosin heads onto the thin actin filaments. For a muscle fiber to contract, the myosin heads must first be activated by ATP (adenosine triphosphate), with each head binding one ATP molecule and hydrolyzing it to ADP (adenosine diphosphate). The energy released from this hydrolysis is transferred to the myosin head, allowing it to bind to the actin filament. Upon binding, energy is released causing the myosin head springs back, dragging the bound actin filament along. This dragging movement, referred to as the power stroke, is the origin of muscle contraction [4]. During contraction, power strokes occur at a rate of ∼17 s^−1^, with myosin propelling actin at a velocity of ∼310 nm/s [19]. Notably, increasing load can accelerate this process [20].

To enable voluntary movement, skeletal muscle is a densely innervated tissue, with motor neurons extending from the central nervous system into the muscle tissue to establish contact with each myofibers at the neuromuscular junction (NMJ) [2,7]. When action potentials are produced, acetylcholine is released and binds to myofibers, depolarizing the cellular membranes and initiating the release of calcium ions within the myofibrils. Calcium ions then bind to the actin filaments, modifying their configuration to expose the myosin-binding sites, which initiate muscle contraction [21]. To meet the considerable metabolic needs created by contractions, skeletal muscle is highly vascularized. Its features a dense capillary network aligned alongside muscle fibers (with around 600 capillaries per millimeter), capturing around 25% of the resting cardiac output, and facilitating efficient exchange of nutrients and oxygen [2].

On top of the dynamic structures formed by myofibrils, skeletal muscle contraction is also sustained by its passive highly organized connective tissue structures. The skeletal muscle extracellular matrix (ECM) forms the supportive network that maintains muscle shape and structural integrity. It enables uniform distribution and force transmission within the muscle and to the tendon, playing an important role in muscle elasticity and allowing the synergic contraction of myofibers during movement [22]. Collagen is the main structural protein in skeletal muscle ECM, accounting for up to 10% of the muscle dry weight, with type I and III being the most abundant. Other ECM proteins include laminin, elastin, fibronectin, and other various proteoglycans [23]. Additionally, by binding nonmatrix molecules such as growth factors, the ECM also creates a dynamic signaling environment essential for muscle growth, regeneration, and homeostasis [24].

Skeletal muscles have an innate remarkable capacity for regeneration, due mostly to the presence of muscle stem cells, also known as satellite cells, in grooves lateral to the longitudinal axis of the muscle fibers [8]. Upon activation, satellite cells can proliferate and differentiate into myoblasts, which will then mature into fully functional myofibers. Therefore, when compared to other closely related tissues such as tendons or ligaments, skeletal muscle stands out by its high repair speed. Within a few weeks after injury, full functional repair can be achieved following a highly orchestrated regeneration process. This process can be divided in 3 main phases [1,25]: (a) a degenerative and inflammatory phase, associated with muscle cell necrosis, hematoma formation, and immune cells invasion; (b) a regenerative phase, consisting of phagocytosis of the necrotic muscle tissue, ECM deposition, and satellite cell activation and proliferation to initiate myofiber regeneration; (c) a remodeling and maturation phase, where functional recovery is reached by including reorganization of the scar tissue, ECM remodeling, maturation of myofibers, angiogenesis, and innervation. For most skeletal muscle injuries, this natural regeneration process is sufficient to allow the tissue to completely recover and regain full functionality [24]. However, when substantial damage is sustained, or in cases of muscle-wasting diseases, this regeneration process is not enough to prevent permanent muscle loss, scar tissue formation, and irreversible loss of function. Several therapeutic strategies are currently being investigated to promote skeletal muscle regeneration, among which SMTE is emerging as a promising approach.

## Conventional SMTE

SMTE is an interdisciplinary field that aims at producing healthy and functional skeletal muscle. The conventional SMTE strategy consists of utilizing bioreactor systems to generate muscle tissue in vitro using scaffolds, human or animal cells, and appropriate growth conditions (i.e., chemical environment and relevant external stimuli) [13,14]. While the intricate interplay between these various parameters makes the production of skeletal muscle tissue in vitro an ongoing challenge, current SMTE approaches provide exciting opportunities for medical applications. To be of interest for clinical use, engineered skeletal muscle needs to fulfill an array of criteria. It needs to be of relevant shapes and sizes, have contractile ability, incorporate itself to the native muscle upon implantation, sustain innervation and vascularization to enable full functionality recovery, etc. In the current state of the SMTE field, most of those specifications remain challenges to be solved. Research mainly focuses on 4 areas: the choice of the scaffold used, of the type of cells cultured, of the stimuli applied, and of the bioreactor in which the tissue maturation happens. Here, an overview of the current strategies explored to address the current limitations of SMTE is proposed.

### Scaffolds

In tissue engineering, scaffolds are temporary structures that support cell growth and 3D development during the tissue developmental stage [1]. To properly guide cells and promote tissue neogenesis, scaffolds need to possess some chemical, mechanical, and structural characteristics that are specific to the target tissue. For SMTE, an ideal scaffold should (a) be biocompatible, (b) biodegrade, with a degradation rate matching the speed of tissue neogenesis, (c) have a stiffness mimicking the one of native skeletal muscle (10 to 20 kPa), and (d) promote myoblast growth, differentiation, and alignment [11]. Examples of existing SMTE scaffolds can be found in Table 1.

**Table 1.** Examples of scaffolds used in SMTE.

Origin	Material	Description	Mechanical properties			Cell interaction		Biodegradability	Ref.
			Manufacturing techniques	Scaffold description	Modulus	Adhesion	Differentiation		
Synthetic	PCL	Polycaprolactone	Electrospinning	Aligned PCL fibers, 0.7 μm diameter	75 MPa	Supports satellite cell adhesion, alignment, and growth	Increased myogenic gene expression at day 4	X	[142]
			3D bioprinting	PCL fiber bundles, 10 μm diameter, plasma treated	25 MPa	Supports myoblast adhesion, proliferation, and alignment	Supports myotube formation, with increased myogenic activity from day 14	No significant degradation after 3 weeks in PBS, 37 °C	[143]
			Freeze-drying	PCL microparticles functionalized with polyPEGMA	10 kPa	Supports myoblast adhesion and promotes proliferation	Supports myotube formation, with up-regulated myogenic markers	X	[144]
	PLGA	Poly(lactic-co-glycolic acid)	Electrospinning	Aligned PLGA fibers	750 MPa	Supports and promotes myoblast adhesion and elongation	Enhances myoblast fusion from 14 d	X	[145]
			Randomly oriented PLGA fibers, with or without added collagen	149 MPa	Promotes myoblast adhesion and elongation	Limited myoblast fusion on PLGA scaffold, but enhances fusion and myogenic gene expression with the addition of collagen	X	[146]
			3D bioprinting	E-jet printed PLGA multilayer scaffold with 50-μm fibrillar gap	X	Enhances myoblast adhesion and proliferation	Supports myotube formation, with increased myogenic gene expression	X	[65]
			Freeze-drying	Plasma-treated PLGA scaffold	X	Supports myoblast adhesion and proliferation in vitro for 48h	Not directly investigated. In vivo experiments show new tissue growth on scaffold 7 d post-implantation	No significant weight loss at 28 d in PBS, 37 °C	[147]
			Hydrogel casting	Microgrooved PLGA hydrogel	X	Promotes myoblast adhesion, enhanced proliferation with wider grooves	Large grove widths and shallow groove depths promote myotube formation and alignment	X	[148]
	PEGDA	Polyethylene glycol diacrylate	3D bioprinting	PEGDA-fibrinogen (145:1) scaffold encapsulating muscle progenitor cells	0.1–1 kPa	Supports cell viability and growth	Supports myotube formation and alignment with spontaneous contraction from day 10	40% degradation in collagenase (0.5 mg/ml, 30 min)	[135,149]
			Hydrogel casting	Photo-crosslinked micropatterned PEGDA hydrogel	33 kPa	Supports myoblast adhesion, and promotes alignment	Supports myotube formation in the axis of the grooves, with myogenic genes up-regulation	X	[150]
Natural	Fibrin	Fibrous ECM protein involved in blood clotting	Electrospinning	Crosslinked fibrin fiber bundles with 0.2% PEO as carrier polymer	17 kPa	Stem cells infiltrate the scaffold and align in the direction of the fibers	Limited success with myogenic differentiation. Absence of multinucleation.	X	[151]
			3D bioprinting	Fibrin hydrogel (3.5% gelatine) with encapsulated myoblasts	15 kPa	90% myoblast viability at day 1. Supports myoblast proliferation and alignment	Thinner constructs promote myotube formation, with enhanced myogenic expression	X	[152]
			Hydrogel casting	Fibrin hydrogel, 5.6 mg/ml density in DMEM	3.3 MPa	Supports satellite cell adhesion, activation, and proliferation	Increased myogenic gene expression and enhanced myotube formation	Complete degradation by day 5 in physiological pH	[153]
	Collagen	Main structural ECM protein. Most abundant protein in mammals	Electrospinning	Calf skin derived type I collagen fibers	38 MPa	Retains 40% of myoblasts upon seeding. Supports proliferation and cell alignment	Supports myoblast formation, displaying cross-striated pattern at 10 d. Spontaneous contraction from day 24	X	[154]
			3D bioprinting	Rat-tail collagen I scaffolds	10–25 kPa	Supports adhesion, proliferation, and alignment of myoblasts	Formation of aligned and densely packed myotubes	X	[155]
			Freeze-drying	Rat-tail collagen I freeze-dried at −60 °C with fibrillogenesis step	33 kPa	Supports myoblast adhesion and alignment	Limited success with myotube formation	70% degradation after 9 h in collagenase solution (50 U/ml)	[156]
				Bovine type I collagen freeze-dried sponges	2.5 kPa	Smaller pore size improved initial cell adhesion, but poorer cell infiltration at the core of the sponges	Smaller pore size associated with higher levels of myogenic gene expression, supporting myotube formation.	X	[157]
			Hydrogel casting	Rat-tail collagen I hydrogel, 4 mg/ml density in PBS	3.7 MPa	Supports satellite cell adhesion, activation, and proliferation	Increased myogenic gene expression, supports myotube formation	No weight decrease at 15 d of culture	[153]
	Alginate	Naturally occurring polysaccharide found in algae. Does not provide mammalian cell adhesion ligands	Electrospinning	Alginate fibers with 2% PEO as a carrier polymer	5 MPa	90% cell viability at days 1 and 7, supports myoblast proliferation	Promotes myoblast elongation, alignment, and myogenic differentiation	X	[158]
			Hydrogel casting	Alginate gels with or without conjugated gelatine and heparin	30–80 kPa	No MSC adhesion on alginate gels, adhesion increases with decreasing % of alginate	Increased myogenic gene expression on alginate–gelatine–heparin gels, with enhanced myotube formation	X	[159]
Natural (ECM)	Plant ECM	Plant-derived decellularized extracellular matrix	Whole decellularized tissue	White asparagus decellularized with detergent treatment	4.9 kPa	Supports myoblast adhesion and proliferation	Supports myotube formation	X	[160]
				Apple slice decellularized with SDS treatment	8–17 kPa	Supports adhesion, infiltration and proliferation	Differentiation not investigated. No visible spontaneous myoblast fusion after 3 weeks in culture	X	[161]
	Non-tissue specific ECM	Decellularized extracellular matrix derived from tissues other than skeletal muscle	Electrospinning	Porcine-derived urinary bladder dECM (UBM) electrospun fibers	6.2 MPa	Supports cell growth, but with lower affinity than on tissue culture plate	Not investigated	35% mass loss after 1 week in PBS, 37 °C	[162]
			Hydrogel casting	UBM hydrogel	X	Supports myoblast adhesion, proliferation, and infiltration	Not investigated	X	[162]
				UBM hydrogel (8 mg/ml)	0.11 kPa	Supports myoblast adhesion and proliferation	Limited myotube formation	Fully degraded in 35 d after in vivo implantation	[57]
				Porcine dermal dECM hydrogel (8 mg/ml)	0.45 kPa	Supports myoblast adhesion and proliferation	Supports radially alignment myotube formation by day 7	Fully degraded in 35 d after in vivo implantation	[163]
	Tissue-specific dECM	Skeletal muscle decellularized extracellular matrix	Electrospinning	Aligned dECM electrospun fibers, with GA crosslinking	648 kPa	Supports myoblast adhesion and proliferation	Promotes myotube differentiation alignment	33% loss of scaffold volume in 10 d in cell culture media	[164]
			3D bioprinting	Multilayer printed porcine skeletal muscle dECM scaffold encapsulating human skeletal muscle cells	10–12 kPa	Over 85% viability, supports cell proliferation	Promotes myotube formation with enhanced myogenic gene expression in vitro	X	[38]
			Whole decellularized tissue	Decellularized rat extensor digitorum longus	X	Supports satellite cell infiltration and proliferation	Supports myotube formation, which can be enhanced by coculture with fibroblasts	X	[24]
			Hydrogel casting	Rabit muscle dECM hydrogel, thermally casted on PLLA nonwoven scaffold	0.25 kPa	Supports myoblast adhesion and proliferation. Cell infiltration into the inner layers reported	Promotes myotube formation, with enhanced myogenic gene expression in vitro. Acceleration of muscle regeneration with increased collagen deposition in vivo.	X	[165]

#### Scaffold composition

To have those desirable properties, SMTE scaffolds can be made from synthetic or natural polymers, including decellularized ECM [22]. Briefly, synthetic polymers possess the advantage of having highly tunable properties, allowing a precise control over their chemical, mechanical, and topological properties. Their structures are established and controlled, and characteristics such as strength, stiffness, and degradation rates are predictable and reproducible, ensuring performance reliability. Synthetic polymers are generally easier to synthesize and process, making them usually more affordable for widespread production than their natural counterparts. However, they lack intrinsic cell adhesion sites, which can lead to low cell attachment and insufficient bioactivity [26]. Bioactivity can be improved through surface coatings or surface functionalization with ECM proteins via physical adsorption or covalent binding [27]. Additionally, synthetic polymers are typically hydrophobic, potentially triggering host immune responses [28], but this limitation can often be mitigated through surface treatments [27]. Nevertheless, concerns persist about their long-term safety due to potential accumulation of their degradation products [28]. Natural polymers are typically nontoxic and highly compatible with biological systems, making them attractive for tissue engineering applications. However, their biological origin gives them an inherent immunogenicity, which can elicit host immune responses if antigenic components are not adequately removed during processing [29]. While their superior bioactivity enhances cell response, their natural origin and production methods lead to high batch-to-batch variability, processing difficulties, and higher production costs, limiting their widespread manufacturing [26]. Natural polymers usually exhibit poorer material properties, such as lower strength, reduced stiffness, and limited shelf life [3]. They also present a higher risk of pathogen contamination, raising concerns about their safety. Despite having better cell affinity than synthetic polymers, natural polymers still lack the complexity of the 3D-extracellular environment, which limits their ability to guide cells toward mature tissue states. ECM-derived scaffolds are a potential solution to address this limitation. Those can be either non-tissue specific (e.g., derived from animal or plant tissues) or tissue specific (e.g., derived from skeletal muscle), the latter offering biochemical cues tailored to the target tissue. Both types are associated with enhanced cellular function, including cell attachment, growth, and differentiation in vitro [1]. However, ECM scaffolds are challenging to manufacture, with limited control over their physiochemical properties [26] and difficulties in scaling to clinically relevant sizes. Additionally, they can trigger inflammation or rejection due to the presence of residual foreign DNA or of nonhuman cell membrane antigens [30]. A standardized decellularization method still needs to be established to ensure that the native genetic material is completely removed while preserving the mechanical and structural properties of the matrix.

#### Manufacturing techniques

Besides the materials used, manufacturing techniques also play an important role in scaffold design to closely imitate the native cellular environment and accurately guide cells toward tissue formation. Importantly, for SMTE, scaffolds should be porous to allow cell penetration, and they should possess an aligned structure mimicking the natural organization of skeletal muscle fibers. To achieve this, various manufacturing techniques can be used, including electrospinning, 3D bioprinting [31], hydrogel micromolding [32], and freeze-drying [11]. Details on those techniques can be found in Box 2. In the case of ECM-derived scaffolds, on top of using ECM slurry to formulate hydrogels, bioinks for 3D bioprinting, or electrospinning solutions, it is also possible to fabricate scaffolds directly by decellularizing whole tissue fragments, which could preserve the native 3D ECM structure [1]. Overall, each technique provides different levels of control over the properties of the final construct and comes with its own set of pros and cons. By carefully selecting the appropriate method and material, the design and functionality of tissue-engineered constructs can be optimized to better mimic natural skeletal muscle.

Box 2.  Common SMTE scaffold manufacturing techniques

**Electrospinning:** Electrospinning is a fiber fabrication process, governed by the application of an electric force that draws charged threads of polymer solutions into nanometric fibers. Various soluble polymers and additives can be used for electrospinning either individually [33] or in blends [34]. This method enables fine control over fiber diameter, morphology, and orientation, making it interesting for creating aligned structures for SMTE scaffolds [3]. However, electrospinning only offers limited control over scaffold porosity, often leading to the formation of dense nanofiber mats that restrict cell infiltration [27]. Strategies to address this limitation include adding sacrificial fibers or microparticles, adjusting the collector’s structure to manipulate density, or introducing post-production treatments, such as ultrasonication or gas foaming, to enhance pore size and improve cell penetration [35]. Additionally, electrospun scaffolds tend to have low mechanical strength and are associated with poor load-bearing properties [36]. This limitation can be mitigated by integrating carbon nanotubes into the fibers, by further arranging the electrospun fibers into weaved, braided, or knitted structures, or by crosslinking the mesh [37].

**3D bioprinting:** 3D bioprinting creates functional structures by layering bioinks combining biomaterials, cells, and support molecules such as growth factors [38]. Common 3D bioprinting methods include inkjet bioprinting, laser bioprinting, and extrusion bioprinting. Inkjet bioprinting uses electromagnetic or piezoelectric force to push our drops of low viscosity bioink onto a substrate, allowing for a fast fabrication of relatively low-cost constructs. However, poor precision on the droplets’ placement limits the resolution of the printed structures. Laser-assisted bioprinting achieves high micrometric precision by vaporizing and propelling bioink with a laser pulse, but this method is associated with long printing times and high operation costs. Finally, extrusion bioprinting employs a mechanical or pneumatic force to expel bioink through a nozzle, producing constructs with high structural integrity and high cell density. A major drawback of this technique is the low cell viability associated, with survival rates as low as 40% [39]. To limit shear stress, prevent cell damage, and thus increase cell survival rates, strategies such as using lower-viscosity or non-Newtonian shear-thinning bioinks, modifying nozzle geometry and size [40], and regulating nozzle and printing chamber temperature can be implemented [41].

**Hydrogel micromolding:** Also known as soft lithography, hydrogel micromolding uses patterned molds to shape hydrogel precursors, which are then cross-linked to retain the mold’s shape [42]. Crosslinking methods include ultraviolet and thermal polymerization. Soft lithography offers high-resolution patterning, enabling the creation of detailed structures. Those microscale topographic cues, often grooves or ridges with optimized dimensions, guide the adhesion, alignment, and differentiation of muscle cells [32]. Unlike photolithography, which directly patterns hydrogel constructs using light beams, soft lithography only requires the master mold to be patterned. Because of this, soft lithography eliminates the systematic need for a clean-room environment, significatively reducing costs of production [27]. While these hydrogels typically exhibit poor mechanical strength and limited resistance to dynamic forces, they can be integrated with stiffer frameworks, such 3D-printed or electrospun structures, enhancing their mechanical properties [32].

**Freeze-drying:** Freeze-drying is a promising technique for fabricating highly porous SMTE scaffolds. It is a dehydration process using ice as a porogen, which involves freezing water-soluble polymer constructs and sublimating ice crystals to create interconnected pores. This technique uses water as a solvent, reducing cytotoxicity risks compared to methods using harsher solvents [11]. Additionally, the resulting high porosity and interconnectivity promotes cell migration and infiltration into the constructs, as well as sufficient nutrient diffusion. Although porosity can be influenced by freezing-rate adjustments, freeze-dried scaffolds often remain heterogeneous, with pore sizes inferior to what is needed for proper cell integration [43].

### Cell types

**Fig. 2.**
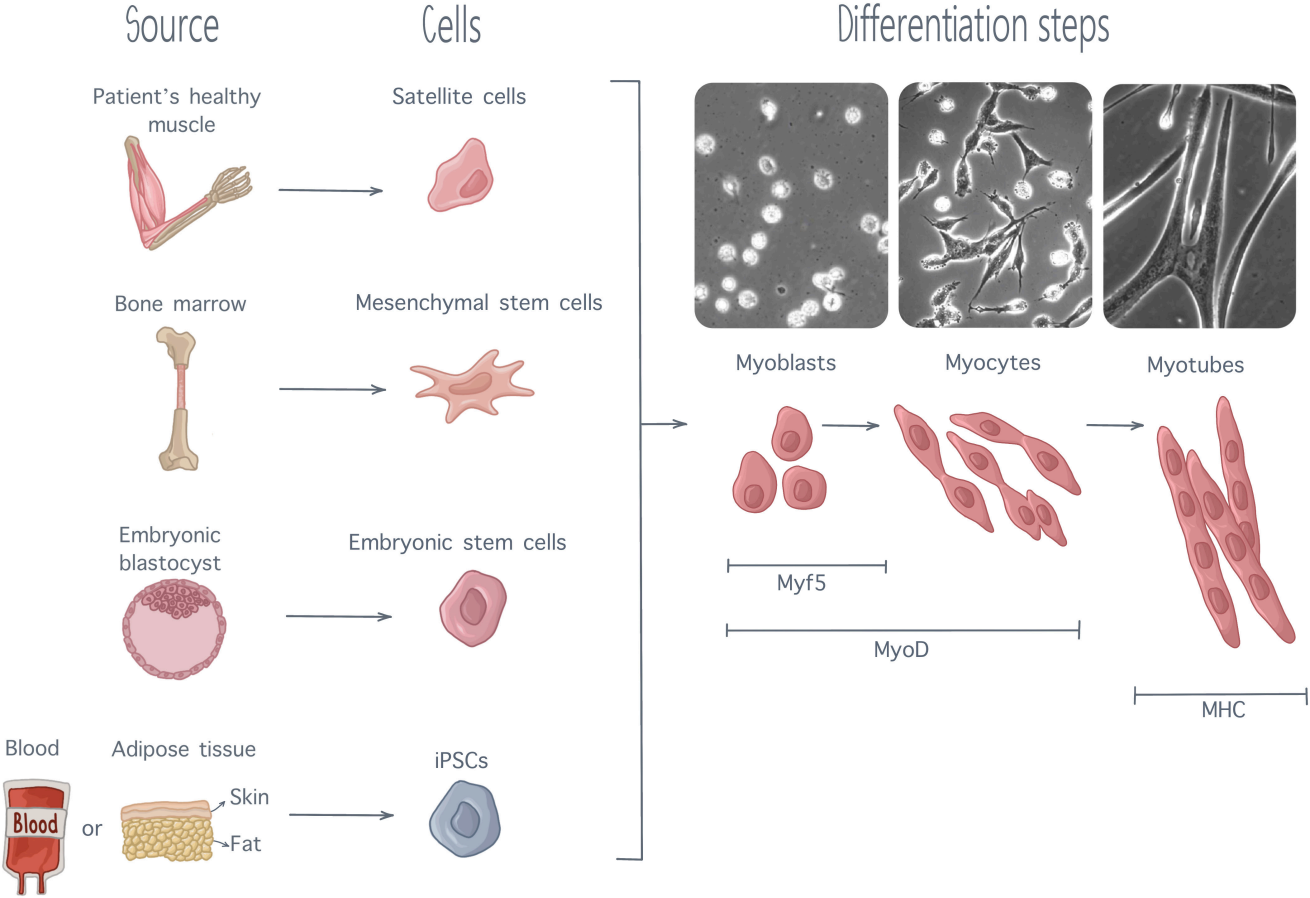
Myogenesis steps, from precursors cells of various sources to fully differentiated myotubes. As differentiation progresses, muscle cells express several transcription factors, including Myf5, MyoD, and MHC expressing subsequent level of maturation. Cell pictures are adapted from [137] with permission from *Development*.

In SMTE, immature cells are supported and guided by scaffolds and external physicochemical cues to undergo myogenic differentiation and form mature, healthy, and contractile muscle tissue. Myogenesis can be broken down in a sequence of steps. Precursor cells differentiate into myoblasts, which start fusing and forming myocytes, which then further fuse and organize themselves into myotubes (Fig. 2) [2]. As they progress down the myogenic differentiation cascade, cells express specific transcription factors, which are distinctive attributes of the current cell state [44]. Put simply, following activation and entry into the cell cycle, precursor cells will start expressing myogenic factor 5 (Myf5), confirming their commitment toward the myogenic lineage. Alongside with Myf5, myoblasts will express a protein called myoblast determination protein 1 (MyoD), which marks myoblast commitment. As myoblasts proliferate and start fusing, Myf5 expression will decrease while MyoD expression will surge. When fully fusing into myofibers, MyoD expression will be replaced by myosin heavy chain protein (MHC) expression, which is considered as a marker of late-stage myoblast differentiation [2,6]. Thus, quantifying those transcription factors is usually done to characterize the progression of myogenic differentiation.

Precursor cell types for SMTE must possess a high proliferative capacity while being able to efficiently differentiate into myotubes [27]. Multiple cell types can be used for clinical applications, including satellite cells, which are stem cells derived directly from the patient’s healthy muscle. Satellite cells are direct precursors of skeletal muscle. They are naturally used for muscle regeneration by the organism and are thus particularly appropriate for SMTE [45]. However, their extraction process is highly invasive, limiting their application to SMTE. Therefore, interest for other types of stem cells with an ability for myogenesis has increased. Stem cell lineages of interest include mesenchymal stem cells (MSCs), embryonic stem cells (ESCs), and induced pluripotent stem cells (iPSCs) [44,46]. MSCs, also known as mesenchymal stromal cells, are multipotent cells with an ability to differentiation into diverse cell types, including cartilage cells, fat cells, bone cells, and muscle cells. They are mainly extracted from the bone marrow (through femur or tibia bone marrow biopsies) or from adipose tissue. Adipose tissue-derived MSCs are easier to obtain, with only limited patient discomfort, and can be expanded in large quantities in vitro; thus, most of the research conducted on MSCs is currently focused on adipose tissue-derived MSCs [45]. Although MSCs can differentiate into muscle cells, their ability for myogenicity is quite limited. It had been established that, in differentiation medium, only 15% of MSCS could differentiate into myocytes [44]. Although this differentiation ability can be enhanced using an appropriate scaffold and external stimulation, this low myogenic ability limits the use of MSCs for SMTE. ESCs are found in the inner cell mass at the early stage of human embryonic development, from the 4th to the 7th day after fertilization [47]. They possess the capacity to differentiate into all cell types existing in the human body and are thus referred to as pluripotent. Although their pluripotency makes them exciting candidates for tissue regeneration, the ethical concerns linked to their embryonic origin strongly limit their usage in research. In recent years, iPSCs have emerged as a promising alternative to overcome the limitation associated with the use of ESCs. These pluripotent cells can be derived from almost every adult tissue, share morphological similarities with ESCs, and possess the ability to proliferate indefinitely [44]. The discovery of the process to convert mature cells back into pluripotent stem cells owed Dr. Shinya Yamanaka and Sir John Gurdon the 2012 Nobel Prize in Physiology or Medicine [48], as iPSCs are extremely promising for regenerative medicine. More precisely for the field of SMTE, it has been shown that iPSCs can generate multinucleated myotubes in 2D culture, while 3D culture into hydrogels allowed iPSCs to differentiate into contractile skeletal muscle tissue constructs [44]. Despite these favorable results, several ethical and safety concerns remain to be addressed surrounding the potential clinical use of iPSCs. A major concern is their tumorigenic potential, which can arise through malignant transformation of differentiated iPSCs or benign teratoma formation from residual partially differentiated cells [49], caused by oncogenes used during the reprogramming process [50]. Additionally, immature iPSCs tend to exhibit deficient DNA damage repair and impaired cell cycle arrest, making them more prone to accumulating genomic abnormalities [51]. A large-scale study, conducted by the International Stem Cell Initiative across 38 laboratories, compared 11 iPSC lines at early and late passages and found that more than 20% displayed increased activity of anti-apoptotic genes after extended in vitro culture [52]. This finding underscores the risk that prolonged iPSC culture may lead to genomic instabilities, further exacerbating their tumorigenic potential. To mitigate these risks, alternative reprogramming factors are being researched as well as protocols to minimize residual undifferentiated cells and targeted elimination of aberrant cells using drug-induced cell suicide [51]. Besides, immunogenicity of iPSCs remains a concern as studies suggest that iPSC-derived cells may elicit immune responses despite being autologous [53]. Traditional immunosuppressive therapies, commonly used to enhance graft tolerance, would be unsuitable for iPSC-based treatments, as suppressing the immune system to prevent rejection could inadvertently further heighten the risk of tumor formation [50]. Given iPSCs’ tumorigenicity, this dual risk presents a challenge for their safe clinical application. Therefore, continuous efforts must be made to accurately detect genetic instabilities and insure homogeneity of cell populations.

For these reasons, stem cell-derived products require strict quality control measures dictated by a strong regulatory framework at all stages of development [50]. At this stage, out of these potential cell sources for SMTE application, none have currently been approved by regulatory bodies for patient usage. Currently, the only stem cells approved by the U.S. Food and Drug Administration (FDA) are hematopoietic progenitor cells, which are blood-forming stem cells that are derived from umbilical cord blood [54]. These stem cells possess a limited ability to support myogenic differentiation when cocultured with myoblast; thus, their interest for SMTE is strongly limited [55]. Additionally, while they have obtained approval for the treatment of blood pathologies, their usage for other purposes remains unauthorized.

While clinical applications require human precursor cells, in current SMTE research, most studies limit themselves to using already partially differentiated muscle cells. One step further down in their commitment toward myogenic phenotype, myoblasts are more affordable, with high proliferation capacity and robustness, and they differentiate easily into myotubes. Consequently, the immortalized mouse myoblast C2C12 cell line is widely used to test scaffolds and stimuli for myotube formation. However, for translation to clinical use, all the research conducted with C2C12 will have to be confirmed with human cell lines. For other applications of SMTE, such a biorobotics or cell-cultured meat production, this translation to human cell lines is not necessarily needed. In this regard, less work remains to be conducted for potential application of SMTE to those fields.

When differentiating into myofibers in vivo, myoblasts receive crucial information from their cellular environment. Information can be delivered by the ECM they are in contact with, by the dynamic mechanical cues of their 3D environment, and by other cells present in their vicinity. A few cell types naturally found in skeletal muscle tissue are known to influence its growth and regeneration, and researchers have been investigating their potential for guiding myoblast differentiation in in vitro cocultures. For instance, macrophages and fibroblast are known to invade the injury site during the muscle repair process. Thus, Venter and Niesler [56] investigated the behavior of myoblasts in in vitro cocultures with those cells. They reported that both macrophages and fibroblasts could significatively promote myoblast proliferation and proliferation. Other important cells when attempting to recreate the native myoblast cellular environment are motor neurons. Motor neurons provide myofibers with the electrical stimulation required to initiate contraction. In vitro 3D cocultures of myoblasts with neural stem cells have been shown to result in the formation of NMJs by Morimoto et al. [57], and the NMJs formed where subsequently used to stimulate the myoblasts to guide their differentiation toward myofibers. While only partially mature myofibers were obtained, contraction of the neuro-muscle constructs was still observed under stimulation. The constructs produced could therefore be attractive candidates for soft robotic bioactuators. Vascular cells, such as epithelial cells, also play an important role in skeletal muscle tissue formation. Indeed, a highly integrated vascular system is key to ensure that the high metabolic demand of skeletal muscle tissue is met. Endothelial cells and muscle progenitor cells share a deeper connection than just their spatial vicinity: They have been shown to mutually promote and regulate one another’s proliferation, migration, and differentiation [58]. However, in vitro cocultures of endothelial cells and myoblasts remain technically challenging, as they both require different growth media composition. While cocultures allow to better encompass and reproduce the complexity of the in vivo myoblast environment, giving promising results in terms of promoting and regulating proliferation and migration, they are also more technically complex to establish and maintain in vitro [6]. Therefore, direct chemical stimulation of engineered skeletal muscle constructs is also being researched. While this would not fully replicate the native myoblast environment, it could still positively impact myoblast development, differentiation, and growth. Details on this approach is given in the next section.

### External stimuli

#### Mechanical stimulation

**Fig. 3.**
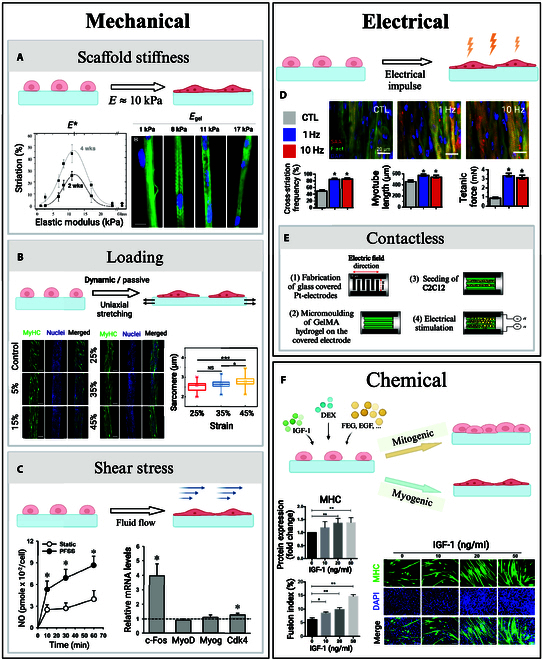
External stimuli in SMTE. (A) Two weeks after seeding myoblasts on collagen-coated substrates, Engler et al. [64] stained for myosin (green) and nuclei (blue). Gels of intermediate stiffness showed notable actin–myosin striation, with an optimal reached for *E* = 12 kPa (scale bar: 20 μm). (B) Morphology of C2C12 cells after stimulation, investigated by Chen et al. [65] Sarcomere lengths increased with the strain ratios (scale bars: 200 μm). (C) Pulsative fluid shear stress doubled nitric oxide production and up-regulated proliferation marker expression in muscle stem cells, presented by Haroon et al. [67]. (D) Khodabukus et al. [68] studied the effects of electrical stimulation on myotube structure. Myobundles stained for α-actinin (red), filamentous actin (green), and nuclei (blue) showed an increase in striation, myotube length, and tetanic force for stimulated constructs. (E) Ahadian et al. [70] presented a contactless electrical stimulator for SMTE. The pH of the culture media remained stable over the time of culture when compared to traditional electrical stimulators, and expression of myogenic factors increased under a 10 V–1 Hz stimulation. (F) Supplementing myoblasts with IGF-1 resulted in enhanced MHC expression and higher fusion indexes after 5 d of culture, presented by Guan et al. [73] (scale bar: 100 μm). Permissions granted where necessary.

Most cells, including those of the musculoskeletal system, are mechanoresponsive [59,60]. While the exact cellular processes involved in mechanotransduction remain to be elucidated, it is known that external mechanical signaling is registered by cells through focal adhesions and a variety of other mechanosensitive structures such as cytoskeleton filaments, mechanically activated ion channels, and myosin motors [61]. These mechanical cues induce cytoskeletal reorganization, driving morphological and metabolic changes [6], muscle growth, protein synthesis, satellite cell activation, and growth factor release. Remarkably, cell differentiation pathways are affected by surface stiffness [62] as focal adhesions allow cells to sense the elasticity of the substrate they are attached to [61]. Engler et al. [63] observed that, when cultured on polyacrylamide gels matching the stiffness of brain tissue (0.1 to 1 kPa), muscle tissue (8 to 17 kPa), and collagenous bone tissue (25 to 40 kPa), MSCs displayed morphological changes and up-regulation of biomarkers associated respectively with neurogenic, myogenic, and osteogenic phenotypes. In the same manner, scaffold stiffness plays an important role in myoblast differentiation toward mature myotubes. Myoblasts cultured on collagen-coated polyacrylamide gels of varied stiffness showed different levels of striation, with well-organized sarcomeres on substrates of intermediate stiffness (8 to 11 kPa) (Fig. 3A). While softer and stiffer substrates (including glass controls) also supported myoblast fusion into multi-nucleated myotubes, only gels possessing a muscle-like stiffness showed advanced levels of myosin striation even at 4 weeks in culture [64]. Typically, higher levels of sarcomere organization are associated with a higher degree of myotube maturation and a higher force production potential. Those findings point toward the existence of an optimal scaffold stiffness to promote maturation of myotube, which would be around 10 kPa, i.e., the stiffness of native skeletal muscle [5]. However, other data suggest that the range of acceptable scaffold mechanical stiffness necessary to promote myotube formation might be wider. Levy-Mishali et al. [61] reported that higher substrate stiffness in the range of 200 to 250 kPa was associated with higher myoblast viability and increased number of myotube formation at 7 d. Those stiffer substrates also resulted in the formation of larger cellular bodies, and they supported higher rates of myotube alignment than the softer gels (4 to 60 kPa). Thus, while scaffold stiffness has been proven to impact myotube viability, morphology, alignment, and sarcomere arrangement, the ideal mechanical properties required to engineer mature functional skeletal muscle still need to be determined.

Besides passive stiffness, skeletal muscle cells also respond to active mechanical stimulation [7]. For instance, Chen et al. [65] reported that, under uniaxial stretching, myoblasts encapsulated in fiber-shaped gelatine methacrylate (GelMA) hydrogel cellular constructs showed higher degrees of cell spreading, elongation, and alignment as well as more pronounced myofiber contractibility (Fig. 3B). A contractibility saturation level was reached for a strain ratio of 35%, which points toward the existence of an optimal stretching degree to favor functional mature myofiber formation. This optimal strain ratio appears consistent with the range of deformation undergone by native skeletal muscle during in vivo contraction (up 40%) [5].

In addition to sensing mechanical loading, skeletal muscle cells also respond to shear stress. Traditional cell culture systems, such as plates or flasks, are static. They only allow for limited gas and nutrient exchange, often causing engineered skeletal muscle tissue to become necrotic at its core. To overcome this limitation, bioreactors with continuous flow of media have been developed. This new generation of culture reactors allow for a more physiological delivery of oxygen and nutrients to the cells, resulting in higher cell survival rates, while also promoting the up-regulation of cell proliferation markers through shear stress-induced cellular mechanisms [66]. Haroon et al. [67] reported that pulsative fluid shear stress loading of muscle stem cells in vitro up-regulated the expression of several genes, including c-Fos and Cdk4, which are known to promote cell proliferation (Fig. 3C). Shear stress also induced higher levels of nitric oxide production, which is associated with enhanced muscle stem cell (MuSC) self-renewal during skeletal muscle regeneration.

#### Electrical stimulation

Sustained contractile activation of skeletal muscle is vital to maintain striated muscle cell viability and function [59]. Electrical stimuli, delivery in vivo by motor neurons, regulates muscle tonus and prevents muscle atrophy [59,68]. In vitro, electrical stimulation has been shown to enhance myogenic differentiation and maturation of engineered striated muscles [69]. Khodabukus et al. [68] studied the effect of intermittent electrical stimulation (1-h stimulation at either 1 or 10 Hz, separated by 7-h rest periods) on the differentiation and maturation of human myobundles (Fig. 3D). They reported that, after 1 week of stimulation, myobundles exhibited an increased number of nuclei, associated with an augmentation of myotube cross-section and length. Cell staining also revealed higher degree of striation, corresponding to more highly organized sarcomeres in stimulated muscle constructs. Increased glucose consumption and higher lactate production indicated a higher cellular metabolic rate, which was associated with a 3-fold increase in tetanic and twitch force output in stimulated myobundles.

While it has been established that electrical stimulation leads to higher degrees of engineered muscle maturation and functionality, currently employed electrical stimulation devices use electrodes that are in direct contact with the muscle constructs or the culture medium in which they are immerged. This contact may cause detrimental effects on the engineered constructs, including hydrolysis of the culture medium, therefore causing bubble formation, pH increases, and temperature elevation of the medium through joule heating. Contamination of the tissue culture environment by the products of electrode corrosion is also made more likely [70]. To overcome those limitations, Ahadian et al. [70] developed a contactless electrical stimulator successfully delivering electrical stimulation to engineered skeletal muscle constructs using glass-covered platinum electrodes (Fig. 3E). They reported that this contactless device allowed to maintain a stable pH of the culture media for up to 6 h of continuous stimulation while also preventing the formation of bubbles. When applying their device to deliver electrical stimulation to muscle myofibers, up-regulated levels of myogenic genes expression were measured, demonstrating higher myotube maturation and contractile abilities.

#### Biochemical stimulation

When progenitor muscle cells terminally differentiate into myotubes in vivo, they receive critical biochemical stimulation from their environment guiding them toward myogenesis. Macrophages, fibroblasts, endothelial cells, and even other myoblasts deliver both structural cues, through cell–cell contact, and chemical cues through the release of growth factors and other chemical stimulants. Inspired by those natural processes, chemical stimulation by delivery of chemical agents to increase proliferation and maturation of engineered skeletal muscle construct is being researched [6]. A variety of growth factors are involved in skeletal muscle tissue development. Most growth factors have a mitogenic impact on muscle precursor cells, which means that they promote cellular division by forcing the cells to reenter the cell cycle [71]. By doing so, they actively block cellular differentiation and are thus boosting cell proliferation but preventing tissue maturation. Such growth factors, for example, include epidermal growth factor (EGF), fibroblast growth factor (FGF), and platelet-derived growth factor (PDGF) [6,71]. Notably, insulin-like growth factor 1 (IGF-1) has been found to have both a mitogenic and a myogenic effect on myoblast. IGF-1 thus promotes both myoblast proliferation and terminal differentiation, setting it apart from other mitogenic growth factors. At the organism level, IGF-1 has been shown to be essential to muscle growth development [72]. Experiments conducted on mice embryo established that the knocking out of IGF-1 gene results in the birth of nonviable pups [71]. At the cellular level, Guan et al. [73] reported that supplementing myoblasts with IGF-1 resulted in enhanced cellular proliferation, with significatively higher mitochondrial DNA quantities measured, and higher degrees of differentiation in a dose-dependent manner (Fig. 3F). The increase in myoblast fusion index was paired with an up-regulated expression of myogenic factors. Other kinds of chemicals have also been investigated as potential stimulant for the development of engineered skeletal muscle constructs. Remarkably, the glucocorticoid dexamethasone (DEX) has been shown to stimulate myoblast differentiation into myotubes. Syverud et al. [74] reported that muscle satellite cell supplementation with DEX exhibited higher rates of myogenic proliferation. Addition of DEX was associated with the formation of denser myotubes with larger cellular bodies.

#### Additional stimulation methods to promote skeletal muscle tissue formation

While mechanical, electrical, and biochemical stimulation remain the most widely used methods for promoting tissue formation and regeneration, other techniques have also shown potential. Notably, magnetic stimulation has emerged as a promising tool for enhancing muscle repair in SMTE approaches [6]. Although still in its early stages, it shows potential across several dimensions. First, magnetic stimulation has been shown to promote muscle regeneration following injury. For instance, using a murine muscle injury model, Stölting et al. [75] reported that external magnetic stimulation significantly reduced inflammatory infiltration and scar formation, prevented post-traumatic muscle atrophy, and promoted hypertrophy. Moreover, it enhanced muscle metabolism and turnover, tripled the expression of muscle-specific markers, and improved functional recovery. The authors concluded that magnetic stimulation supported both muscle and nerve regeneration by enhancing muscle-nerve cross-talk and promoting the maturation of NMJs. Second, magnetic fields can be used to guide and manipulate the 3D organization of engineered tissues. This is particularly advantageous for promoting muscle cell alignment, which is critical to achieve high functionality of the engineered muscle. Yamamoto et al. [76] demonstrated that magnetically labeled C2C12 myoblasts could be aligned around hollow fibers using external magnetic fields. This setup enabled the formation of dense, multilayered muscle constructs, highlighting the utility of magnetic stimulation through cell labeling to form large-scale skeletal muscle constructs [77]. Beyond in vitro applications, magnetically labeled cells also hold promise for direct in vivo tissue engineering approaches. Studies have shown that such cells could be injected and subsequently guided to injury sites using external magnetic fields, facilitating targeted regeneration of musculoskeletal tissues such as bone, cartilage, and skeletal muscle, in a minimally invasive manner [78]. For instance, Nakabayashi et al. [79] delivered magnetically labeled MSCs into a rat model of muscle injury and observed enhanced cell proliferation within 72 h under magnetic targeting. Histological and biomechanical analyses confirmed improved repair outcomes in the treated muscles. Collectively, these findings highlight the versatility and therapeutic potential of magnetic stimulation as a noninvasive strategy in skeletal muscle regeneration and tissue engineering.

Finally, as the different types of stimulation reviewed here have shown great promise for tissue engineering of skeletal muscle constructs, ways of combining those stimulation methods together to further promote muscle growth are now being investigated [6]. For instance, Liao et al. [80] have reported that a combined electromechanical stimulation (5% cyclic strain at 1 Hz from 2 d post-differentiation with 20 V at 1 Hz from 7 d post-differentiation) induced significant increase in myogenic factor expression in myoblasts. Synchronized dual stimulation resulted in the formation of myotubes with a higher degree of striation. In brief, while the results reported in the literature are promising, additional work needs to be conducted to establish optimal stimulation settings for all parameters, and progress also needs to be made to efficiently deliver the combined stimuli to the construct for optimal engineered skeletal muscle tissue maturation and functionality.

### Bioreactor systems

To deliver the different stimuli mentioned above, researchers have been focusing on the development of bioreactor systems. Bioreactors play a crucial role in tissue engineering as they provide a controlled environment supporting the metabolic needs of tissue constructs [81]. To properly support tissue constructs of any kind, a bioreactor must possess some key fundamental characteristics. It needs to be easy to sterilize and able to maintain sterility over the duration of tissue culture. All the materials used for the bioreactor must be cytocompatible, and their corrosion when in contact with tissue culture medium must be minimal. It should provide appropriate biochemical conditions, which include gases and nutrient concentrations, pH, and temperature, and allow for control of the operating conditions over time [59]. Additionally, a bioreactor should enable to monitor the culture conditions, either through built-in captors or through visual input [82]. As bioreactors should aim to mimic the native cellular environment, bioreactors for SMTE must ideally also enable the delivery of mechanical or electrical stimulation to the muscle constructs [83]. As skeletal muscles exist in high stress environments and possess considerable metabolic needs, bioreactor design for SMTE is particularly challenging.

**Fig. 4.**
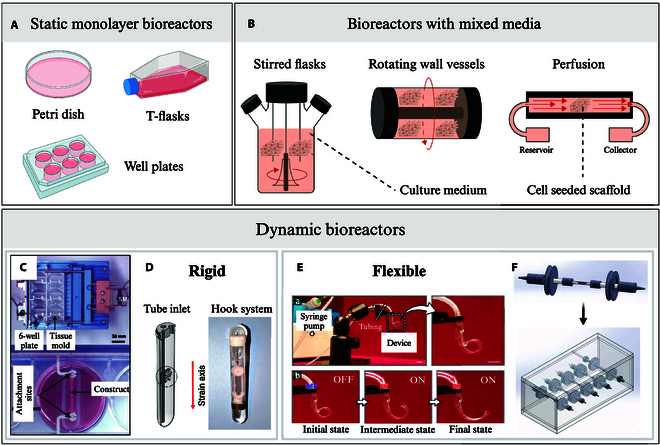
Examples of SMTE bioreactors. (A) Static monolayer culture bioreactors: petri dishes, tissue culture flasks (T-flasks), and well plates. (B) Bioreactors with mixed media: stirred flasks, rotating wall vessels, perfusion [138]. Rigid bioreactors: (C) mechanical cell stimulator (modified 6-well plate) by Powell et al. [139] and (D) the MagneTissue bioreactor (custom tube-inlet fitted into a modified falcon tube) by Heher et al. [140]. Flexible bioreactors: (E) pneumatic soft robotic in vitro platform for cell culture by Paek et al. [110] and (F) perfused flexible tubing lines hosting one bioconstruct each, fitted into a Plexiglas chamber, by Quarta et al. [141]. Permissions granted where necessary.

Over the years, many bioreactor systems have been developed for engineering skeletal muscle, with various degrees of complexity (Fig. 4). The simplest bioreactor systems are static monolayer culture bioreactors, which include T-flasks, multi-well plates, and petri dishes. They are economical, simple to use, and easy to sterilize, but they require manual handling for medium exchange. Their major limitation is that they can only support tissue with a limited thickness (around 100 μm) due to insufficient gas exchange via surface aeration and limited diffusion-driven nutrient transfer [59,84], leading to nonuniform tissue formation with preferential cell growth around scaffold edges [85]. To improve mass transfer and allow the culture of thicker tissue constructs, a range of bioreactor systems with continuously mixed media have been developed [59]. Stirred flasks, where cell-seeded scaffolds are suspended in stirred culture medium, are an accessible and relatively simple example of such dynamic bioreactor systems [86]. Turbulent mixing is facilitated by a magnetic stir bar at the bottom of the flask, generating convective forces that enhance nutrient distribution, while temperature and oxygen levels are regulated by an incubator in which the whole setup is placed [85]. While this method allows for higher cell seeding density, more uniform cell distribution compared to static models [85], and improved overall cell viability [59], the stirring setup creates an uneven shear stress distribution leading to nonhomogeneous culture conditions. This can lead to the formation of a dense superficial cell layer, limiting nutrient diffusion to the tissue core [86]. Offering more stable fluid flow than stirred flasks, rotating wall vessel bioreactors, developed by NASA in the 1990s [87], freely suspend scaffolds in media between 2 concentrical cylinders. The outer cylinder rotates, while the inner cylinder of a rotating wall vessel remains stationary, creating a microgravity-like environment that promotes media mixing with a laminar fluid flow [85]. This reduced shear stress while minimizing diffusional limitations of nutrients and waste, therefore supporting higher cell proliferation with lower apoptosis compared to static cultures. However, frequent scaffold collisions with the bioreactor wall may cause cell injury, disrupting attachment and matrix deposition [86]. Finally, perfusion bioreactor systems offer superior mass transfer by continuously circulating media through scaffold pores, promoting homogeneous cell distribution with high seeding efficiencies [85]. The resulting shear forces mechanically stimulates cells, benefiting skeletal muscle tissue formation [66]. However, excessive shear stress can lead to cell washout and can hinder ECM deposition and result in poor cytoskeletal organization [86]. Additionally, the removal of perfusion conditions upon in vivo implantation risks causing rapid tissue necrosis, posing challenges for clinical translation of constructs produced this way [59].

Both the static and dynamic systems mentioned above are built with hard and rigid chambers, often using modified cell culture well plates as the base component. While they are relatively easy to build and operate, rigid bioreactors display very limited performances when it comes to delivering mechanical stimuli as they are usually limited to uniaxial stretching [88]. For skeletal muscle, where mechanical stimulation plays such an important role in tissue maturation, this could limit the functionality of the constructs produced. To overcome this limitation and deliver physiologically relevant multiaxial stresses to the constructs, soft flexible bioreactors have been developed in recent years. Table 2 recapitulates rigid and flexible SMTE bioreactors developed along with their key characteristics. Unlike rigid traditional bioreactors, those new systems are equipped with soft flexible chambers that can undergo a wider variety of multiaxial deformation such as twisting, bending, stretching, and compression [88]. Materials suggested to construct soft chambers allowing this versatility in movement tend to be more permeable to oxygen than materials traditionally used in hard rigid bioreactors. Thus, soft chambers passively improve gas exchange, ensuring a more adequate delivery of oxygen to the engineered skeletal muscle constructs. However, soft materials can rupture more easily, which makes leak proofness and sterility harder to maintain, especially under load-bearing conditions [88]. Additionally, to accurately monitor the mechanical and biochemical conditions in soft bioreactors, flexible integrated sensors must be developed, adding another level of complexity to bioreactor design [89]. While soft chambers are facing those technical challenges and thus remain an emerging area, progress is steadily being made, with increasing efforts and innovations pointing toward their transformative potential. The growing interest in soft, flexible bioreactors is particularly encouraging for SMTE, as such systems not only address the challenges outlined above but also offer an exciting opportunity to bridge tissue engineering with the advancing field of biorobotics.

**Table 2.** Current bioreactor systems for SMTE. The presence of an * indicates that the mechanical stimulation is due to shear stress produced by the presence of a liquid flux.

Reactor type	Description	Stimulation			Cell line	Date	Ref.
		Mechanical	Electrical	Magnetic			
Rigid	Perfused commercially available “CellCo” culture system	+ *	-	-	Embryonic avian muscle cells	1998	[166]
Rigid	Covered glass spinner flasks	+ *	-	-	C2C12 myoblast	2001	[167]
Rigid	Modified 6-well plate	+	-	-	Primary human skeletal muscle cells	2002	[139]
Rigid	Rectangular Plexiglas culture chamber	+	+	-	C2C12 myoblasts	2007	[82]
Rigid	Modified tissue culture container	+	-	-	Human muscle precursor cells	2008	[168]
Rigid	Scaffold wrapped around silicon tubing into rigid container	+	+	-	C2C12 myoblasts	2009	[80]
Rigid	Polycarbonate chambers perfused with laminar flow	+ *	-	-	C2C12 myoblasts	2009	[169]
Rigid	Modified 6-well plate	-	+	-	C2C12 myoblasts	2010	[170]
Rigid	Plexiglas culture chamber	+	-	-	C2C12 myoblasts	2010	[171]
Rigid	Polycarbonate hollow fibers	-	-	+	C2C12 myoblasts	2012	[76]
Rigid	Silicon chambers into modified 5-well plates	+	-	-	Murine muscle-derived cells	2012	[172]
Rigid	Fibrin ring scaffolds in modified falcon tubes	+	-	-	C2C12 myoblasts	2015	[140]
Rigid	Perfusion seeding bioreactor with 2 glass columns	-	+	-	Primary mouse myoblasts	2015	[173]
Rigid	Actuation chamber hosting a modified well plate	+	+	-	Human adipose-derived stem cells	2016	[97]
Rigid/flexible	Rigid reactor bow containing 4 flexible chambers	+ *	-	-	Human muscle stem cells	2017	[141]
Rigid	3D-printed perfused chamber	+ *	-	-	C2C12 myoblasts	2019	[174]
Rigid	Hydraulic chamber with PDMS membrane	+	-	-	None	2021	[175]
Flexible	Curling pneumatic device with integrated culture chamber	+	-	-	Human uterine muscle cells	2021	[110]
Rigid	Sterile culture compartment clamped onto an actuator	+	-	-	C2C12 myoblasts	2021	[110]
Rigid	Metallic chamber equipped with a mobile grip system	+	-	-	C2C12 myoblasts	2022	[176]
Flexible	Silicon chamber with 2 metallic frames in an incubator	+	-	-	C2C12 myoblasts	2024	[177]

### Alternatives to conventional SMTE

The conventional SMTE approaches discussed earlier rely on a combination of scaffolds, cells, and bioreactors to generate functional muscle tissue. To simplify this complex process, alternatives have emerged to bypass one of these key elements, leading to scaffold-free, cell-free, and in vivo engineering methods, each offering distinct advantages and challenges. Additionally, advancements in microfluidic technologies, such as organoids and organ-on-chip systems, provide innovative platforms for studying muscle physiology and disease. This section briefly explores these alternative approaches, highlighting their potential applications and limitations in advancing skeletal muscle regeneration.

#### Scaffold-free, cell-free, and stimulation-free approaches

Scaffold-free approaches in SMTE eliminate the need for an external 3D matrix, relying instead on direct cell delivery [90]. For SMTE, one of the most common methods involves the direct intramuscular injection of cells to the damaged area [7]. While this technique is straightforward and has shown some promising preliminary results [91], it faces considerable challenges such as poor cell retention, low survival rates, and immune rejection of the transplanted cells [7]. To improve retention, alternative scaffold-free strategies have been explored, including through the formation of cell sheets or cell aggregates. The coculture of satellite cells and fibroblasts that self-assemble into cylindrical muscle constructs that can later be implanted is a promising approach for SMTE [92]. However, while they offer better structural integration, such approaches are constrained by the time required for the formation of ECM structures robust enough for integration—often over 4 weeks—and the limited construct size due to nutrient diffusion constraints [92].

In contrast, cell-free approaches, also known as in situ tissue engineering, leverage acellular biomaterial scaffolds to induce endogenous regeneration. By engineering precise biophysical and biochemical cues, these scaffolds can guide host cell recruitment, activation, proliferation, and differentiation [7]. This method offers several advantages, including faster and simpler fabrication, streamlined delivery, and off-the-shelf availability due to the elimination of cell culture requirements [14]. Additionally, regulatory barriers are often lower, accelerating clinical translation [7]. However, a key challenge lies in ensuring uniform cell infiltration throughout the scaffold, particularly at its core, which is critical for achieving consistent and controlled tissue regeneration.

A further alternative to conventional tissue engineering is in vivo tissue engineering, which can be seen as a “stimulation-free approach”. It involves seeding scaffolds with cells immediately before transplantation to minimize cell manipulation, thus preserving cell functionality and simplifying the preparation process [7]. However, transplanted cells remain vulnerable to low viability, retention issues, and immune rejection within the host environment and the success of this method depends on scaffold properties and implantation conditions to enhance cell survival and integration [7].

#### Organoids and organ-on-chips

Organoids and organ-on-chips (OoCs) represent an extension of conventional tissue engineering, integrating microfluidics to create highly controlled microenvironments that better replicate physiological conditions. These systems operate at the microscale, with dimensions typically confined to around 100 μm to ensure appropriate flow dynamics [93]. While both organoids-on-chips and organs-on-chips merge tissue engineering with microfluidics, their concepts and focuses differ slightly. Organoids-on-chips rely on the self-organization of stem cells to form 3D tissue clusters, which are then cultured on microfluidic chips to support long-term maintenance. In contrast, organs-on-chips do not rely on self-organization; instead, they are fully microengineered platforms incorporating microfluidic channels designed to replicate tissue interfaces and physiological forces present in living tissue. Applied to SMTE, OoCs provide advanced platforms for studying muscle physiology and pathology by closely mimicking the structural organization, functional properties, and regenerative capacity of native skeletal muscle [94]. Consequently, their primary applications lie in drug discovery and disease modeling, where they serve as powerful tools for high-throughput testing with minimal reagent and compound use [93,95]. Additionally, these platforms possess interesting potential in the field of robotics, particularly in the development of microbiorobots. Specifically, they can be employed as testing grounds for microdrug delivery robots [96]. Although the scope of biorobotics discussed in this review excludes a detailed exploration of this application, OoCs remain highly promising technologies that continue to push the boundaries of biomedical research. For a more in-depth analysis, we refer interested readers to previously published reviews [93,94,97].

### Challenges in clinical translation of SMTE strategies

Up to this point, this review has primarily examined the individual building blocks of engineered skeletal muscle, including scaffolds, cells, and growth environment, highlighting their respective advantages and limitations. However, as SMTE is ultimately driven by the clinical need to repair large muscle defects, the successful integration of these components into a cohesive and functional tissue is essential. This includes ensuring appropriate donor–host integration and long-term survival of the engineered muscle [98]. At this higher level of abstraction, new challenges emerge, which are not tied to any single component but rather arise from the complex interplay between them. Therefore, these challenges often cannot be resolved by modifying building blocks independently but instead require more complex strategies that account for the full system and its host environment. As a result, many of these issues remain unresolved and continue to represent major barriers to clinical translation.

One of these central challenges toward functional clinical translation is the vascularization of engineered constructs. Skeletal muscle has colossal metabolic needs, requiring an abundant supply of nutrients and oxygen to function and survive [2]. Without vascular networks, construct size is severely limited by diffusion constraints, risking necrosis in central regions and rendering such constructs inherently unsuitable for large defect repair. In vitro strategies to achieve vascularization include coculturing muscle cells with endothelial cells, optimizing scaffold properties, such as incorporating hollow channels, and supplementing with angiogenic factors [99]. While these approaches show promise, work remains to fully achieve vascularization and overcome the size limitations of constructs. Even when viable vascular networks are achieved in vitro, rapid integration with the host’s circulation post-transplantation is necessary to prevent hypoxia-induced cell dead. This remains a major translational challenge [98]. Another major hurdle is innervation, and more specifically, the rapid integration of the engineered muscle with the host’s neuromuscular system. Without timely innervation, grafts will remain nonfunctional in the short term and will risk atrophy over time [98]. Lastly, immune rejection of constructs also threatens host integration. The recipient’s immune system may identify the engineered tissue as foreign and attack it. Using nonimmunogenic scaffolds and autologous cells can mitigate this risk [100], but this approach comes with its own set of drawbacks. Cell harvesting procedures can be invasive, and the regenerative potential of harvested cells often declines during extended in vitro culture [101]. In addition, prolonged graft growth time may allow the injury site to begin healing, allowing scar tissue formation, which would further complicate functional integration [102].

Regulatory hurdles must also be addressed to enable the clinical translation of SMTE approaches. The standardization of engineered constructs and of procedural guidelines remains an obstacle. Universal standards remain to be established, complexified by the interdisciplinarity of the field. Additionally, the financial cost of bringing SMTE therapies to market is a considerable burden. While exact costs are uncertain, pharmaceutical companies typically spend over $850 million to develop and commercialize new products [103]. Given the complexity of SMTE—encompassing multiple components such as cells, scaffolds, bioreactors, and growth factors, all of which may require individualized production methods—the associated costs are expected to be even higher. Despite these challenges, the tissue engineering market is projected to grow by about 10% over the next decade [104], reflecting ongoing advancements and continuous interest. While SMTE primarily aims at repairing large muscle defects, other promising applications like drug discovery, disease modeling, cell-cultured meat, and biorobotics may reach practical use sooner, sustaining enthusiasm for the field.

## Advanced Robotics and Muscle Tissue Engineering

### Advanced robotics applied to SMTE

In musculoskeletal tissue engineering, it is widely acknowledged that mechanical stimulation plays a crucial role in the growth and maturation of tissues. To deliver the active mechanical loading required to promote tissue growth, simple bioreactor systems have traditionally been used to apply uniaxial stretching and compression [88]. Several commercially available bioreactors, such as the Ebers TC-3 [105], the BioTense [106], or the CellScale MCTX [107], operate on this principle. While these systems effectively provide basic mechanical stimulation, they are inherently limited by their lack of sensing and adaptive control [108]. This poses a challenge as the mechanical properties of engineered tissues evolve over time due to tissue remodeling through scaffold degradation and ECM deposition [6]. Therefore, a fixed set of mechanical stimulation parameters that may be appropriate at the beginning of culture may become inadequate as the tissue matures. To address this, Smith et al. [108] integrated a linear actuator into an autonomous robotic stimulator that dynamically adjusts the applied force in response to changes in tissue stiffness. This ensures a consistent relative force throughout the culture period, enhancing the reliability and effectiveness of mechanical stimulation.

Although the incorporation of adaptive control methods in robotic bioreactors represents a promising advancement in tissue engineering, most systems remain limited to linear mechanical stimulation, therefore failing to accurately replicate the complex mechanical environment native tissue experiences in vivo. In the body, tissues are subjected to a combination of tensile, compressive, torsional, and shear stresses, which are difficult to recreate using conventional stimulation methods. As a result, there is growing interest in the development of more advanced biomimetic systems that can better simulate physiological conditions. One such system is the computer-controlled benchtop bioreactor developed by Altman et al. [109]. This bioreactor enables the application of complex combinations of translational and rotational mechanical strains to 3D cell-seeded matrices and allows users to select between perfusion and shear flow settings. Their findings demonstrated a 2-fold increase in the cross-sectional cell density of human bone marrow stromal cells grown with their bioreactor setup compared to statically cultured tissue. Another example, drawing from the field of soft robotics, is the pneumatic soft robotic constrictor for tissue culture developed by Paek et al. [110]. This system was used to apply dynamic bending and compression forces to various cell types, including smooth muscle cells, resulting in morphological changes and enhanced cell alignment in stimulated cultures. However, while these systems do go one step forward on mechanical complexity, they still fall short of fully replicating native tissue conditions.

**Fig. 5.**
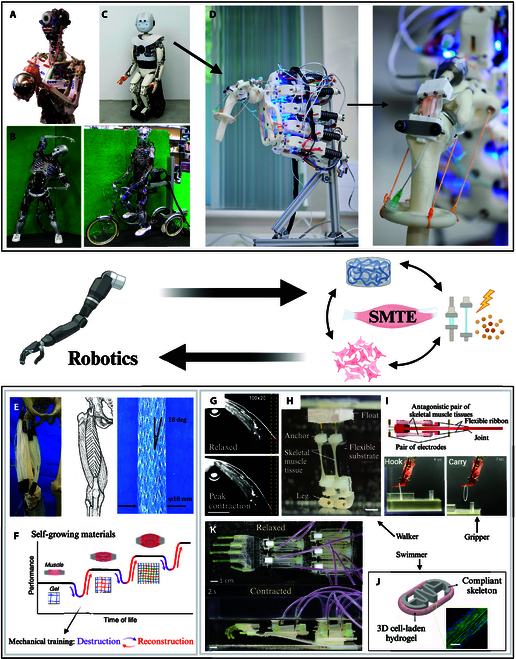
Complementary relationship between advanced robotic systems and SMTE. Musculoskeletal humanoid robots, like (A) “Eccerobot” [112], (B) “Kenshiro” [111], or (C) “Robody” [113], have the potential to serve as bioreactor platforms for SMTE. (D) Adapted shoulder from “Robody” hosting a tendon tissue construct, presented by Mouthuy et al. [114]. In return, skeletal muscle is a source of inspiration for artificial robotic systems. (E) Comparison between human quadriceps muscle structure and multifilament musculoskeletal McKibben muscle robot developed by Kurumaya et al. [117]. (F) Self-growing double-network hydrogels inspired by skeletal muscle mechanical training presented by Matsuda et al. [118]. Skeletal muscle can also be used as an actuator. Example of current muscle-actuated systems include (G) a 2D cantilever ridged system by Sun et al. [121], (H) a walker by Kinjo et al. [125], (I) a gripper by Morimoto et al. [124], and (J) a swimmer by Guix et al. [128]. (K) An 18-cm biohybrid hand, capable of moving individual fingers and manipulating objects by Ren et al. [129]. Permissions granted where necessary.

One of the most promising approaches for achieving biomimetic mechanical stimulation involves musculoskeletal humanoid robots. These systems are designed to closely imitate human body proportions, skeletal structures, muscle arrangements, and natural joint motion [88], making them ideal candidates for replicating the mechanical environment required for advanced tissue engineering applications. With degrees of freedom and actuator arrangements modeled to match human anatomical characteristics, the nature and magnitude of forces experienced by musculoskeletal robots’ joints can be deemed physiologically relevant. Examples of musculoskeletal robots include “Kenshiro” (Fig. 5B), created by the Inaba group in Japan [111], “Eccerobot” (Fig. 5A), developed through a cross-European collaboration [112], and “Robody” (Fig. 5C), developed by the German start-up Devanthro GmbH [113]. With their ability to closely mimic the human musculoskeletal system, these robots could be proposed as bioreactor platforms for growing tissue grafts for clinical use.

In a recent pilot study, Mouthuy et al. [114] demonstrated the potential of using humanoid robots as platforms for musculoskeletal tissue engineering. A “Robody” musculoskeletal shoulder was equipped with a flexible bioreactor chamber containing a tendon tissue construct (Fig. 5D). This chamber was attached on one side to the robot’s humerus and on the other to a tendon-driven actuator, designed to replicate the size and position of the supraspinatus rotator cuff tendon. The study examined the effects of low-intensity (11 N) and high-intensity (45 N) loading regimens, focusing on low-range abduction–adduction movements. After 2 weeks of daily 30-min loading cycles, the low-intensity regimen showed increased immunofluorescence, indicating enhanced cell proliferation and viability. Although the dynamic culture conditions did not yield significantly superior results than the static ones, this study still demonstrated the feasibility of using musculoskeletal humanoid robots for tissue engineering applications and provided an incentive to keep investigating this strategy. Next steps contemplated in this study include investigating the effects of varied loading regimes by modulating the intensity, range of movement, and duration of the experiments, along with exploring other scaffold materials and cell types. This proof-of-concept study, while centered on a specific robotic platform, opens the door for further exploration using other musculoskeletal humanoid systems like “Kenshiro” or “Eccerobot”. Additionally, while this work focused on tendon constructs, the approach investigated could be adapted and translated to other types of musculoskeletal tissues such as skeletal muscle tissue. In the long term, humanoid bioreactor strategies could yield several benefits, including the creation of enhanced in vitro musculoskeletal models for preclinical research, the production of functional tissue grafts for patients, as well as support and inspiration for the advancement of novel robotic systems [114].

### SMTE applied to robotics

Skeletal muscle possesses a range of fascinating and unique characteristics, such as mechanical compliance, an exceptional power-to-weight and force-to-weight ratio, fine motor control, and even self-repair and adaptability through training [5]. Its ability to handle both delicate precision work and powerful tasks makes it a compelling model for innovation in engineering and robotics [115].

#### Bioinspired artificial muscles

Artificially mimicking natural actuation systems provides many opportunities for developing the next generation of robotic systems [116]. By studying skeletal muscle structure, contraction, and regeneration, and by investigating materials possessing muscle-like properties, SMTE could be a great source of inspiration for the field of biorobotics. Research on robotic systems imitating muscle actuation is actively being undertaken [117]. Efforts are being focused on all scales of skeletal muscle function, ranging from the reproduction of the general anatomy and organization of muscle systems to the replication of intrinsic biological processes. Indeed, the general filament-like structure of whole skeletal muscles has been used to design musculoskeletal-driven robots able to perform human-like motions. For instance, Kurumaya et al. [117] developed a prototype lower-limb robot equipped with thin and soft multifilament actuators inspired by skeletal muscle structure. Their system encompasses McKibben actuators, possessing an elasticity and a compliance akin to the one of native muscle, arranged onto anatomically correct bone-like structures (Fig. 5E). The artificial muscles built this way generated similar contracting forces and ratios as native muscles and could be simply arranged into diverse bundle shapes to reproduce natural muscle structures. By doing so and replicating the muscle placement and redundancy found in a human leg, their prototype robot demonstrated a range of motion in both the knee and ankle that closely matched that of a human. On the microscopic scale, skeletal muscles also possess some key characteristics that are of crucial interest for the development of soft robots. This includes muscles’ ability to self-repair, as well as their ability to grow and reshape themselves to adjust to mechanical overloading. Inspired by such muscle metabolic processes, Matsuda et al. [118] developed self-growing double-network hydrogels able to gain strength and mass under repetitive mechanical stimulation (Fig. 5F). Such materials could be used to create adaptive robotic systems that could remodel themselves to more efficiently perform tasks. The development of bioinspired artificial muscles, and more generally of anatomically relevant artificial musculoskeletal systems, for robotics is a very active area of research that is already being translated outside of academic laboratories to industrial settings. For example, Elysium Robotics [119] is developing dielectric elastomer materials to produce electrostatic actuators with comparable performance to that of human muscles, and the German start-up Devanthro [120] commercializes « Robodies » equipped with tendon-like actuation systems, which imitate the human musculoskeletal system.

#### Biohybrid robots

In addition to producing useful knowledge on skeletal muscle physiomorphology, inspiring new biomimetic artificial actuator designs, SMTE can also be employed to produce biological actuators. Using engineered skeletal muscle constructs as actuators would present multiple advantages over traditional mechanical motors. For instance, bundles of muscle fibers within a construct could be selectively activated through an innervation-like stimulation system to allow dynamic control and precise adjustment of force generation. Soft muscle-actuated biological robots could thus demonstrate higher adaptability capacities, allowing one singular system to perform a variety of activities, from delicate tasks like microsurgery operations to strength-intensive actions like unloading heavy machinery [5]. Unlike conventional actuators, which often require complex control systems to modulate power output, muscle-based actuators could inherently adjust their contractile force, enabling a single system to transition seamlessly between different operational demands. Besides, as mentioned previously, native skeletal muscle possesses the capacity to self-repair. While extensive defects remain challenging to tackle for the in vivo repair machinery, engineered biological actuators gifted with a similar or even enhanced ability for regeneration would revolutionize the field of robotics by creating resilient systems capable of withstanding and recovering from damage. This would be particularly advantageous for robots deployed in extreme or remote environments, such as space exploration, underwater missions, or disaster response scenarios, where maintenance and repair are challenging. Furthermore, skeletal muscles’ innate ability to grow and reshape themselves as a result of mechanical overloading could be exploited to develop actuators able to autonomously adapt to tasks with different power requirements [118]. This could be of particular interest for applications in prosthetics, for instance, as an integrated biohybrid actuator could gradually strengthen in response to increased usage, improving long-term efficiency and adaptability for the user. Thus far, SMTE approaches have aimed at facilitating the formation of aligned skeletal muscle tissue to enhance the electrical and mechanical characteristics of the constructs produced. For biorobotics, additional considerations must be made. Specifically, skeletal muscle constructs must then be designed with an appropriate structural layout, allowing them to ensure seamless functional integration into robotic devices [5]. Recent advancements in SMTE have enabled the development of several proof-of-concept robotic systems actuated with engineered skeletal muscle structures. These systems typically consist of a soft structural framework, often made with elastomers or hydrogels, combined with contractile, mature muscle constructs [115]. Upon controlled contraction, the muscle tissue deforms the structural framework, producing movement in specific sections of the robotic system.

The feasibility of muscle-actuated biohybrid robots currently depends on the system scale. For small and light systems, 2D actuators are sufficient to produce the limited forces required to generate movement. These systems are relatively simple to engineer as they only require the culture of a thin 2D muscle tissue layer (i.e., with a thickness below 100 μm) directly onto the deformable substrate [5]. When integrated in cantilever-based systems, contraction of the 2D muscle layer can result in bending of the whole structure, thus generating a 3D deformation. An example of such systems can be found in the work conducted by Sun et al. [121] on skeletal muscle thin films. By culturing C2C12 cells onto printed fibronectin lines of various widths (20, 50, 100, and 200 μm) and spacings (10, 20, and 30 μm), they were able to produce engineered skeletal muscle tissue with consistent contractile properties inducing deformation at the millimeter-scale deformations (Fig. 5G). Such systems could be adapted to generate flagellar motion, serving as a propulsion mechanism for microscale biohybrid robots [122]. These microrobots could find applications in the biomedical field, particularly for targeted drug delivery in localized pathologies. Applications could include delivering therapeutics directly to cancer tumors, minimizing globalized side effects, or treating biofilm infections, where their small size and propulsive thrust would enable them to breach the film barriers and deliver drugs precisely to the affected area [123].

For larger biorobotics systems with dimensions exceeding the millimeter scale, 2D actuators become insufficient to achieve relevant deformations. In these cases, 3D skeletal muscle constructs are required to generate greater forces [115]. Various 3D muscle-actuated robotic systems have been developed, with designs often inspired by living organisms. They are traditionally grouped by the type of movement they can perform, i.e., gripping, walking, or swimming [124]. Examples of such robots are given in Fig. 5 and are discussed below. Grippers are built with the purpose to pick-up, lift, and manipulate objects [115]. Like soft robotic grippers, skeletal muscle-actuated gripper systems are particularly promising for handling delicate or fragile object, where conventional robotic grippers may exert excessive force. In recent years, numerous proof-of-concept skeletal muscle-actuated grippers have been developed, an interesting example of which being the one built by Morimoto et al. [124]. Their approach relied on the use of selective contractions from a pair of antagonist skeletal muscle constructs, which allowed to generate a large actuation amplitude (approximately 90° rotation of the robotic articulation) while preventing issues caused by the spontaneous shrinkage of tissues. Tissue shrinkage usually happens throughout the course of culture as intrinsic traction forces intensify. This shrinkage significantly reduces the length of skeletal muscle tissues, leading to impaired contraction. While increasing the stiffness of the culture substrate might help prevent tissue shrinkage, it also results in impaired function as the substrate then becomes too tough to be deformed by contraction of the muscle tissue. As a result, achieving both large actuation and long-term functionality in a biohybrid robot remains challenging, highlighting once again the importance of the scaffold’s mechanical properties. As a rule, a scaffold needs to be soft enough to be deformed by the contraction of the skeletal muscle tissue while being strong enough to prevent tissue shrinkage and the loss of function associated with it. Finding inspiration in biological systems, Morimoto et al. [124] proposed an innovative solution to this problem by using an antagonistic pair of skeletal muscle constructs to actuate their systems. Alternated contraction of each muscle enabled movements, allowing the robot to lift and carry objects, while compensating tension between the opposing muscles prevented long-term shrinkage (Fig. 5I). Walkers and swimmers are 2 other categories of robots sharing a common goal, i.e., autonomous locomotion, via 2 different approaches. Recent progress has led to proof-of-concept devices that showcase the potential of such systems. For instance, Kinjo et al. [125] developed a 2-legged biohybrid robot (Fig. 5H), each incorporating a built-in skeletal muscle actuation system, able to perform both forward-stop motions and fine turning motion. This is of particular interest as previously

described walker and swimmer systems can usually only turn while moving forward, resulting in considerable turning trajectories and raising the concern that such robots might not be employable in confined crowded spaces where fine turning motion is needed. Similarly, swimmers are designed to autonomously evolve and operate within cell culture media. Many versions of swimming biorobots have also been developed, often inspired by natural biological systems such as sting rays [126] or sperm cells [127]. Some designs are more original, like the skeletal muscle-actuated swimmer supported by a serpentine spring skeleton proposed by Guix et al. [128]. The spring skeleton provided both structural integrity and mechanical self-stimulation to the cellular construct, allowing it to outperform other muscle-based swimmers with a maximum velocity of 800 μm/s (equivalent to 3 body lengths per second) (Fig. 5J). At a bigger scale than microscale biohybrid robots, muscle-actuated swimmers also possess potential for applications in miniaturized transport, such as targeted drug delivery in the biomedical field.

To date, most muscle-actuated systems, including those presented here thus far, have been restricted to just a few centimeters in size, and limited to relatively simple design, typically requiring only a single joint. These limitations severely restrict their potential applications and prevent such systems from generating complex movements or actuating large structures. However, a recent breakthrough by Ren et al. [129] has demonstrated a promising approach to scaling up muscle-actuated designs. Leveraging the relationship between contractile force and engineered muscle length, long muscle constructs were arranged in parallel to form bundle units, effectively increasing overall force output. This approach enabled the successful actuation of an 18-cm biohybrid hand, capable of selectively moving individual fingers and manipulating objects (Fig. 5K). This innovative design framework opens new possibilities for constructing larger, more sophisticated biohybrid robots. Additionally, it presents opportunities in other fields, such as drug-testing models that assess contractility as a performance metric [129].

Another key aspect of the development of biohybrid muscle-actuated robotics is the integration of an appropriate control mode. Indeed, while effective actuation is essential, precise control of actuator behavior is also fundamental to enabling functional and adaptive robotic movement [130]. For skeletal muscle-based biohybrid robots, control systems must ideally be noninvasive, biocompatible, and accurate. The most common method to date is electrical stimulation, typically delivered via wired electrodes placed in the extracellular space. While relatively simple to implement, this approach suffers from several drawbacks, including nonuniform electric fields, limited spatial precision, and invasiveness due to the need for embedded electrodes [131]. To address these limitations, alternative nonelectrical control strategies are being explored, including optical [132], magnetic [131], chemical [133], and neural-based methods [127]. To permit optical control, skeletal muscle cells are genetically modified to express light-sensitive ion channels, enabling contraction in response to light-emitting diode (LED) pulses [131]. For example, Raman et al. [132] developed a modular light-controlled muscle actuator, which, when integrated into a biohybrid robot, enabled both forward motion (1.3 body lengths/min) and controlled 2D rotation. While offering precise control, this technique faces limitations, including poor light penetration through tissue, and the need for genetic modification, which remains technically challenging and may raise safety concerns for clinical applications [131]. Magnetic control is a promising alternative as it is similarly wireless, can penetrate deep in tissues, and allows for precise spatial control enabling navigation through complex environments where direct physical tethering or electrical stimulation would be infeasible [131]. For instance, Liu et al. [131] developed biohybrid magnetic microswimmers by coating microalgae with magnetic nanoparticles and using magnetic fields to guide them toward singular muscle fibers. Upon near-infrared irradiation, the particles induced a mild local temperature increase of about 5 °C, triggering targeted muscle contraction without causing tissue damage. Chemical control is a less developed alternative but holds potential, especially for biomedical applications requiring autonomous navigation. By mimicking chemotactic behavior, biohybrid robots could respond to specific molecular gradients and targets, which could be produced by inflamed tissues or tumor microenvironment. An example of such a system is the one by Sun et al. [133], which was designed by combining asymmetric claws with a carbon nanotube-based myocardial layer to enable caterpillar-like motion. Remarkably, the robot adjusted its speed in response to varying ion concentrations in the surrounding medium, suggesting the feasibility of environmentally responsive movement. Finally, efforts are being made to replicate natural muscle control via neural interfaces. Although still in early stages, this approach could enable biologically integrated control architectures. Aydin et al. [127], for example, developed a 2-tailed flagellar swimmer driven by neuromuscular units, demonstrating proof-of-concept neural actuation. However, optical stimulation was still required to activate nerve endings, meaning the system remained dependent on externa inputs and possessed increased complexity rather than replacing existing control modes.

The concepts and systems presented in this part highlight how the fields of SMTE and robotics have a complementary relationship, each offering valuable insights that can benefit the other. By working more closely together, they could unlock even greater potential for innovation. While proof-of-concept systems in these areas show promise for the development of cutting-edge robotic technologies, challenges remain in translating these advancements from the laboratory to real-world applications.

## Limitations, Future Perspectives, and Concluding Remarks

Several challenges must still be addressed to scale up and implement these proof-of-concept skeletal muscle actuators into functional robotics systems. Many of these challenges overlap with those encountered in clinical applications, yet the constraints of each application diverge. Despite differences, the shared key issues—particularly vascularization—underscore the interconnection between the 2 disciplines. Progress in one area can directly inform and inspire advancements in the other, making collaboration between SMTE and biorobotics research highly beneficial.

### Vascularization and innervation

As with clinical applications, vascularization remains a critical challenge in scaling engineered skeletal muscle for biorobotics. Skeletal muscles require a continuous and abundant supply of nutrients and oxygen [2]; thus, the current lack of vasculature in engineered constructs greatly limits their size. While clinical strategies ultimately rely on integration with the host vascular network, biorobotics faces the additional challenge of sustaining muscle function entirely in vitro. Indeed, unlike in clinical applications, where host integration is a goal, biohybrid actuators require fully autonomous self-sustaining vascular-like systems able to sustain nutrient exchange over extended periods without biological incorporation. A possible approach to address this limitation involves harnessing natural vascularization processes by implanting constructs in vivo, allowing the host’s biological response to drive angiogenesis, before explanting constructs to integrate them in robotics systems [5]. However, ethical concerns obviously prevent this method from being scaled up, since the use of animals as living incubators is not sensible. Other strategies, similar to the one used for clinical applications, such as coculturing muscle and endothelial cells or supplementing angiogenic factors [99], are more sensible but still require extensive work to achieve the desired functional vascular networks. As it directly limits the size and functionality on engineered muscle constructs, vascularization can be considered the most pressing issue on which research efforts should be concentrated. Without effective nutrient and oxygen delivery systems, even small-scale constructs cannot be sustained for extended periods, making further advancements impractical. Innervation, or the lack thereof, follows closely in terms of research priority as it is key to ensure muscle contractibility and the sustained functionality necessary for both clinical and robotics applications. While in clinical contexts ensuring integration with the host’s neuromuscular system is essential to restore function and prevent atrophy, in biorobotics, however, the challenge lies in achieving precise and controllable muscle actuation without biological nerve integration. To circumvent the need for biological innervation, some innovative approaches have been proposed, such as using microelectrodes or optogenetics to selectively activate specific bundles of myofibers [115]. However, these technologies are still in their early days and require substantial development before they can be applied to complex robotic systems.

### Cell source

For engineered skeletal muscle constructs to be effectively scaled up and applied to robotics, having a reliable and abundant cell source that can be easily expanded in vitro is crucial. While clinical applications may prioritize autologous cells to reduce risks of immune rejection, biorobotics requires a cell source that ensures high expansion potential, cost-effectiveness, and ethical viability. Establishing such a cell source must be addressed early, as it dictates the feasibility of scaled-up production. Previous work has shown that muscle constructs derived from primary skeletal muscle cells generate significantly greater contractile forces compared to those made from myoblast cell lines. Primary cells also exhibit higher excitability, requiring less stimulation to induce contraction [134]. However, there is a major limitation to the use of primary cells: They have limited capacity for expansion in vitro [5]. Additionally, harvesting these cells from animals on a large scale for biorobotics raises major ethical concerns. Alternative approaches would be to differentiate muscle cells from either embryonic or induced pluripotent stem cells [44]. Yet, this strategy also presents major drawbacks. The use of ESCs is ethically prohibited in many contexts and iPSCs remain prohibitively expensive. Consequently, identifying a cost-effective and ethical cell source for mass production remains an ongoing challenge.

### Long-term in vitro culture of skeletal muscle constructs

Unlike with clinical applications where engineered constructs are designed to integrate the host environment, constructs destined for robotics systems must remain viable and functional over extended durations in vitro. Long-term maintenance strategies, essential for future real-world applications, should be refined in parallel and adapted to advances made toward the other crucial challenges. Achieving this goal poses several challenges, as it requires maintaining precise conditions, such as optimal temperature, gas exchange, pH levels, and a consistent nutrient supply, all while keeping the sterility of the culture environment. Sterility is especially critical as engineered muscle tissue constructs lack an immune system, which makes them highly vulnerable to infections from bacteria, viruses, and fungi [135]. One promising solution to maintain sterility overtime is the use of flexible bioreactor chambers, like the one proposed by Mouthuy et al. [114]. These soft, flexible chambers would allow for a full range of motion without hindering the force generation of muscle contractions while maintaining sterility by ensuring that the constructs are completely isolated from external contaminants. While the feasibility of this approach for SMTE remains to be investigated, preliminary results from tendon tissue constructs have shown considerable potential of those chambers for musculoskeletal tissue engineering. Another potential strategy would involve the use of more resilient cells, able to thrive in harsher environments than the ones required for mammalian cell lines. Insect muscle cells, for instance, offer an intriguing alternative. Baryshyan et al. [136] demonstrated that such cells could be isolated from *Manduca sexta* eggs and were less metabolically demanding than murine myoblasts. These cells were engineered into muscle bundles capable of self-repair, were able to survive for months without nutrient supply, and could generate contractile stresses. Developing a reliable insect muscle cell line that could be expanded and differentiated in vitro could thus simplify the maintenance of engineered skeletal muscle constructs and eliminate the need for primary mammalian tissue sources.

In conclusion, while substantial work remains to be done, the potential applications of SMTE are vast and impactful. As discussed, the prospects for biorobotics are especially promising, with engineered muscle offering a potential breakthrough in the development of responsive, biologically integrated systems. Continued interdisciplinary collaboration between tissue engineers and roboticists will be crucial in overcoming these hurdles and unlocking the full potential of biohybrid robotic systems. In the medical field, these constructs could lead to the production of personalized functional tissue grafts for patients and to the creation of advanced in vitro musculoskeletal models for preclinical research in disease modeling and drug development. Beyond medical and robotic applications, SMTE also plays a pivotal role in cultured meat production, offering an ethical and sustainable alternative to conventional meat. SMTE is an interdisciplinary field that unites biology, engineering, and material science, generating valuable new knowledge leading to innovations. Given its wide range of applications and potential to address critical challenges in multiple industries, continued research in this area is not only worthwhile but also essential.

## Data Availability

No new data were created or analyzed during this study. Data sharing is not applicable to this article.

## References

[1] Philips C, Terrie L, Thorrez L. Decellularized skeletal muscle: A versatile biomaterial in tissue engineering and regenerative medicine. Biomaterials. 2022;283: Article 121436. 35248912 10.1016/j.biomaterials.2022.121436

[2] Mukund K, Subramaniam S. Skeletal muscle: A review of molecular structure and function, in health and disease. Wiley Interdiscip Rev Syst Biol Med. 2020;12(1): Article e1462. 31407867 10.1002/wsbm.1462 PMC6916202

[3] Kang MS, Lee SH, Park WJ, Lee JE, Kim B, Han D-W. Advanced techniques for skeletal muscle tissue engineering and regeneration. Bioengineering. 2020;7(3):99. 32858848 10.3390/bioengineering7030099 PMC7552709

[4] Frontera WR, Ochala J. Skeletal muscle: A brief review of structure and function. Calcif Tissue Int. 2015;96(3):183–195. 25294644 10.1007/s00223-014-9915-y

[5] Duffy RM, Feinberg AW. Engineered skeletal muscle tissue for soft robotics: Fabrication strategies, current applications, and future challenges. Wiley Interdiscip Rev Nanomed Nanobiotechnol. 2014;6(2):178–195. 24319010 10.1002/wnan.1254

[6] Mueller C, Trujillo-Miranda M, Maier M, Heath DE, O’Connor AJ, Salehi S. Effects of external stimulators on engineered skeletal muscle tissue maturation. Adv Mater Interfaces. 2021;8(1):2001167.

[7] Carnes ME, Pins GD. Skeletal muscle tissue engineering: Biomaterials-based strategies for the treatment of volumetric muscle loss. Bioengineering. 2020;7(3):85. 32751847 10.3390/bioengineering7030085 PMC7552659

[8] Yoshimoto Y, Oishi Y. Mechanisms of skeletal muscle-tendon development and regeneration/healing as potential therapeutic targets. Pharmacol Ther. 2023;243: Article 108357. 36764462 10.1016/j.pharmthera.2023.108357

[9] Cieza A, Causey K, Kamenov K, Hanson SW, Chatterji S, Vos T. Global estimates of the need for rehabilitation based on the global burden of disease study 2019: A systematic analysis for the global burden of disease study 2019. Lancet. 2021;396(10267):2006–2017. 33275908 10.1016/S0140-6736(20)32340-0 PMC7811204

[10] World Health Organization. Musculoskeletal health. [accessed 10 Oct 2024]. https://www.who.int/news-room/fact-sheets/detail/musculoskeletal-conditions

[11] Kozan NG, Joshi M, Sicherer ST, Grasman JM. Porous biomaterial scaffolds for skeletal muscle tissue engineering. Front Bioeng Biotechnol. 2023;11:1245897. 37854885 10.3389/fbioe.2023.1245897 PMC10579822

[12] Edouard P, Reurink G, Mackey AL, Lieber RL, Pizzari T, Järvinen TAH, Gronwald T, Hollander K. Traumatic muscle injury. Nat Rev Dis Primers. 2023;9(1):56. 37857686 10.1038/s41572-023-00469-8

[13] Sander IL, Dvorak N, Stebbins JA, Carr AJ, Mouthuy P-A. Advanced robotics to address the translational gap in tendon engineering. Cyborg Bionic Syst. 2022;2022:9842169. 36285305 10.34133/2022/9842169 PMC9508494

[14] Reid G, Magarotto F, Marsano A, Pozzobon M. Next stage approach to tissue engineering skeletal muscle. Bioengineering. 2020;7(4):118. 33007935 10.3390/bioengineering7040118 PMC7711907

[15] Menciassi A, Laschi C. Biorobotics. In: *Robotics: Concepts, methodologies, tools, and applications*. Hershey (PA): IGI Global Scientific Publishing; 2014. p. 1613–1643.

[16] Biorobotics definition and synonyms—Robotics24 Glossary. [accessed 12 Feb 2025] https://robotics24.net/blog/glossary/biorobotics/

[17] Orzechowski A. Sarcopenia and the inflammatory cytokines. In: *Sarcopenia*. Amsterdam (The Netherlands): Elsevier; 2021. p. 139–157.

[18] Knöll R, Buyandelger B, Lab M. The sarcomeric Z-disc and Z-discopathies. Biomed Res Int. 2011;2011(1):69628. 10.1155/2011/569628 PMC3199094 22028589

[19] Takagi Y, Farrow RE, Billington N, Nagy A, Batters C, Yang Y, Sellers JR, Molloy JE. Myosin-10 produces its power-stroke in two phases and moves processively along a single actin filament under low load. Proc Natl Acad Sci USA. 2014;111(18):E1833–E1842. 24753602 10.1073/pnas.1320122111 PMC4020102

[20] Tang W, Ge J, Unrath WC, Desetty R, Yengo CM. Cardiomyopathy mutations impact the actin-activated power stroke of human cardiac myosin. Biophys J. 2021;120(11):2222–2236. 33864791 10.1016/j.bpj.2021.04.007 PMC8390809

[21] Kuo IY, Ehrlich BE. Signaling in muscle contraction. Cold Spring Harb Perspect Biol. 2015;7(2): Article a006023. 25646377 10.1101/cshperspect.a006023 PMC4315934

[22] Qazi TH, Mooney DJ, Pumberger M, Geißler S, Duda GN. Biomaterials based strategies for skeletal muscle tissue engineering: Existing technologies and future trends. Biomaterials. 2015;53:502–521. 25890747 10.1016/j.biomaterials.2015.02.110

[23] Gholobova D, Terrie L, Gerard M, Declercq H, Thorrez L. Vascularization of tissue-engineered skeletal muscle constructs. Biomaterials. 2020;235: Article 119708. 31999964 10.1016/j.biomaterials.2019.119708

[24] Urciuolo A, De Coppi P. Decellularized tissue for muscle regeneration. Int J Mol Sci. 2018;19(8):2392. 30110909 10.3390/ijms19082392 PMC6121250

[25] Stratos I, Mittlmeier T. Muscle, Ligament and tendon regeneration. In: Steinhoff G, editor. *Regenerative medicine—From protocol to patient*. Cham: Springer International Publishing; 2016. p. 349–366.

[26] Iyer H, Galiano R. Bioinductive scaffolds—Powerhouses of skeletal muscle tissue engineering. Curr Pathobiol Rep. 2017;5:279–288.

[27] Jana S, Levengood SKL, Zhang M. Anisotropic materials for skeletal-muscle-tissue engineering. Adv Mater. 2016;28(48):10588–10612. 27865007 10.1002/adma.201600240 PMC5253134

[28] Reddy MSB, Ponnamma D, Choudhary R, Sadasivuni KK. A comparative review of natural and synthetic biopolymer composite scaffolds. Polymers. 2021;13(7):1105. 33808492 10.3390/polym13071105 PMC8037451

[29] Gomes M, Azevedo H, Malafaya P, Silva S, Oliveira J, Silva G, Sousa R, Mano J, Reis R. Chapter 6—Natural polymers in tissue engineering applications. In: van Blitterswijk C, Thomsen P, Lindahl A, Hubbell J, Williams DF, Cancedda R, de Bruijn JD, Sohier J, editors. *Tissue engineering*. Burlington: Academic Press; 2008. p. 145–192.

[30] Badylak SF, Gilbert TW. Immune response to biologic scaffold materials. Semin Immunol. 2008;20(2):109–116. 18083531 10.1016/j.smim.2007.11.003 PMC2605275

[31] Thangadurai M, Ajith A, Budharaju H, Sethuraman S, Sundaramurthi D. Advances in electrospinning and 3D bioprinting strategies to enhance functional regeneration of skeletal muscle tissue. Biomater Adv. 2022;142: Article 213135. 36215745 10.1016/j.bioadv.2022.213135

[32] Volpi M, Paradiso A, Costantini M, Świȩszkowski W. Hydrogel-based fiber biofabrication techniques for skeletal muscle tissue engineering. ACS Biomater Sci Eng. 2022;8(2):379–405. 35084836 10.1021/acsbiomaterials.1c01145 PMC8848287

[33] Zhang Y, Zhang Z, Wang Y, Su Y, Chen M. 3D myotube guidance on hierarchically organized anisotropic and conductive fibers for skeletal muscle tissue engineering. Mater Sci Eng C. 2020;116: Article 111070. 10.1016/j.msec.2020.111070 32806237

[34] Pham-Nguyen O, Son YJ, Kwon TW, Kim J, Jung YC, Park JB, Kang BJ, Yoo HS. Preparation of stretchable nanofibrous sheets with sacrificial coaxial electrospinning for treatment of traumatic muscle injury. Adv Healthc Mater. 2021;10(8):2002228. 10.1002/adhm.202002228 33506655

[35] Ameer JM, Pr AK, Kasoju N. Strategies to tune electrospun scaffold porosity for effective cell response in tissue engineering. J Funct Biomater. 2019;10(3):30. 31324062 10.3390/jfb10030030 PMC6787600

[36] Zulkifli MZA, Nordin D, Shaari N, Kamarudin SK. Overview of electrospinning for tissue engineering applications. Polymers. 2023;15(11):2418. 37299217 10.3390/polym15112418 PMC10255387

[37] Khorshidi S, Solouk A, Mirzadeh H, Mazinani S, Lagaron JM, Sharifi S, Ramakrishna S. A review of key challenges of electrospun scaffolds for tissue-engineering applications: Challenges regarding electrospun scaffolds: A review. J Tissue Eng Regen Med. 2016;10(9):715–738. 25619820 10.1002/term.1978

[38] Choi Y-J, Jun YJ, Kim DY, Yi HG, Chae SH, Kang J, Lee J, Gao G, Kong JS, Jang J, et al. A 3D cell printed muscle construct with tissue-derived bioink for the treatment of volumetric muscle loss. Biomaterials. 2019;206:160–169. 30939408 10.1016/j.biomaterials.2019.03.036

[39] Bishop ES, Mostafa S, Pakvasa M, Luu HH, Lee MJ, Wolf JM, Ameer GA, He TC, Reid RR. 3-D bioprinting technologies in tissue engineering and regenerative medicine: Current and future trends. Genes Dis. 2017;4(4):185–195. 29911158 10.1016/j.gendis.2017.10.002 PMC6003668

[40] Wang J, Cui Z, Maniruzzaman M. Bioprinting: A focus on improving bioink printability and cell performance based on different process parameters. Int J Pharm. 2023;640: Article 123020. 37149110 10.1016/j.ijpharm.2023.123020

[41] Malekpour A, Chen X. Printability and cell viability in extrusion-based bioprinting from experimental, computational, and machine learning views. J Funct Biomater. 2022;13(2):40. 35466222 10.3390/jfb13020040 PMC9036289

[42] Leijten J, Seo J, Yue K, Trujillo-de Santiago G, Tamayol A, Ruiz-Esparza GU, Shin SR, Sharifi R, Noshadi I, Álvarez MM, et al. Spatially and temporally controlled hydrogels for tissue engineering. Mater Sci Eng R Rep. 2017;119:1–35. 29200661 10.1016/j.mser.2017.07.001 PMC5708586

[43] O’Brien FJ, Harley BA, Yannas IV, Gibson L. Influence of freezing rate on pore structure in freeze-dried collagen-GAG scaffolds. Biomaterials. 2004;25(6):1077–1086. 14615173 10.1016/s0142-9612(03)00630-6

[44] Yu D, Cai Z, Li D, Zhang Y, He M, Yang Y, Liu D, Xie W, Li Y, Xiao W. Myogenic differentiation of stem cells for skeletal muscle regeneration. Stem Cells Int. 2021;2021:8884283. 33628275 10.1155/2021/8884283 PMC7884123

[45] Tang X, Daneshmandi L, Awale G, Nair LS, Laurencin CT. Skeletal muscle regenerative engineering. Regen Eng Transl Med. 2019;5(3):233–251. 33778155 10.1007/s40883-019-00102-9 PMC7992466

[46] Ostrovidov S, Shi X, Sadeghian RB, Salehi S, Fujie T, Bae H, Ramalingam M, Khademhosseini A. Stem cell differentiation toward the myogenic lineage for muscle tissue regeneration: A focus on muscular dystrophy. Stem Cell Rev Rep. 2015;11(6):866–884. 26323256 10.1007/s12015-015-9618-4

[47] National Research Council, Institute of Medicine Committee on the Biological, Biomedical Applications of Stem Cell Research. Embryonic stem cells. In: *Stem cells and the future of regenerative medicine*. Washington (DC): National Academies Press; 2002.

[48] Gurdon J. The developmental capacity of nuclei taken from intestinal epithelium cells of feeding tadpoles. J Embryol Exp Morphol. 1963;10:622–640. 13951335

[49] Lee AS, Tang C, Rao MS, Weissman IL, Wu JC. Tumorigenicity as a clinical hurdle for pluripotent stem cell therapies. Nat Med. 2013;19(8):998–1004. 23921754 10.1038/nm.3267 PMC3967018

[50] Moy A, Kamath A, Ternes S, Kamath J. The challenges to advancing induced pluripotent stem cell-dependent cell replacement therapy. Med Res Arch. 2023;11(11):4784. 38188933 10.18103/mra.v11i11.4784 PMC10768945

[51] Zhong C, Liu M, Pan X, Zhu H. Tumorigenicity risk of iPSCs in vivo: Nip it in the bud. Precis Clin Med. 2022;5(1):pbac004. 35692443 10.1093/pcmedi/pbac004 PMC9026204

[52] The International Stem Cell Initiative. Screening ethnically diverse human embryonic stem cells identifies a chromosome 20 minimal amplicon conferring growth advantage. Nat Biotechnol. 2011;29(12):1132–1144. 22119741 10.1038/nbt.2051 PMC3454460

[53] Liu X, Li W, Fu X, Xu Y. The immunogenicity and immune tolerance of pluripotent stem cell derivatives. Front Immunol. 2017;8:645. 28626459 10.3389/fimmu.2017.00645 PMC5454078

[54] U.S. Food and Drug Administration. Important patient and consumer information about regenerative medicine therapies. [accessed 14 Dec 2024] https://www.fda.gov/vaccines-blood-biologics/consumers-biologics/important-patient-and-consumer-information-about-regenerative-medicine-therapies

[55] Xynos A, Corbella P, Belmonte N, Zini R, Manfredini R, Ferrari G. Bone marrow-derived hematopoietic cells undergo myogenic differentiation following a Pax-7 independent pathway. Stem Cells. 2010;28(5):965–973. 20333749 10.1002/stem.418

[56] Venter C, Niesler C. A triple co-culture method to investigate the effect of macrophages and fibroblasts on myoblast proliferation and migration. BioTechniques. 2018;64(2):52–58. 29571282 10.2144/btn-2017-0100

[57] Morimoto Y, Kato-Negishi M, Onoe H, Takeuchi S. Three-dimensional neuron–muscle constructs with neuromuscular junctions. Biomaterials. 2013;34(37):9413–9419. 24041425 10.1016/j.biomaterials.2013.08.062

[58] Christov C, Chrétien F, Abou-Khalil R, Bassez G, Vallet G, Authier FJ, Bassaglia Y, Shinin V, Tajbakhsh S, Chazaud B, et al. Muscle satellite cells and endothelial cells: Close neighbors and privileged partners. Mol Biol Cell. 2007;18(4):1397–1409. 17287398 10.1091/mbc.E06-08-0693 PMC1838982

[59] Rangarajan S, Madden L, Bursac N. Use of flow, electrical, and mechanical stimulation to promote engineering of striated muscles. Ann Biomed Eng. 2014;42(7):1391–1405. 24366526 10.1007/s10439-013-0966-4 PMC4069203

[60] Kamkin A. Kiseleva I. Mechanosensitivity of cells from various tissues. In: *Mechanosensitivity in cells and tissues*. Moscow (Russia): Academia Publishing House Ltd.; 2005.

[61] Levy-Mishali M, Zoldan J, Levenberg S. Effect of scaffold stiffness on myoblast differentiation. Tissue Eng A. 2009;15(4):935–944. 10.1089/ten.tea.2008.0111 18821844

[62] Yang Y, Wang K, Gu X, Leong KW. Biophysical regulation of cell behavior—Cross talk between substrate stiffness and nanotopography. Engineering. 2017;3(1):36–54. 29071164 10.1016/J.ENG.2017.01.014 PMC5653318

[63] Engler AJ, Sen S, Sweeney HL, Discher DE. Matrix elasticity directs stem cell lineage specification. Cell. 2006;126(4):677–689. 16923388 10.1016/j.cell.2006.06.044

[64] Engler AJ, Griffin MA, Sen S, Bönnemann CG, Sweeney HL, Discher DE. Myotubes differentiate optimally on substrates with tissue-like stiffness. J Cell Biol. 2004;166(6):877–887. 15364962 10.1083/jcb.200405004 PMC2172122

[65] Chen X, du W, Cai Z, Ji S, Dwivedi M, Chen J, Zhao G, Chu J. Uniaxial stretching of cell-laden microfibers for promoting C2C12 myoblasts alignment and myofibers formation. ACS Appl Mater Interfaces. 2020;12(2):2162–2170. 31856565 10.1021/acsami.9b22103

[66] Carrier RL, Papadaki M, Rupnick M, Schoen FJ, Bursac N, Langer R, Freed LE, Vunjak-Novakovic G. Cardiac tissue engineering: Cell seeding, cultivation parameters, and tissue construct characterization. Biotechnol Bioeng. 1999;64(5):580–589. 10404238 10.1002/(sici)1097-0290(19990905)64:5<580::aid-bit8>3.0.co;2-x

[67] Haroon M, Klein-Nulend J, Bakker AD, Jin J, Seddiqi H, Offringa C, de Wit GMJ, le Grand F, Giordani L, Liu KJ, et al. Myofiber stretch induces tensile and shear deformation of muscle stem cells in their native niche. Biophys J. 2021;120(13):2665–2678. 34087215 10.1016/j.bpj.2021.05.021 PMC8390894

[68] Khodabukus A, Madden L, Prabhu NK, Koves TR, Jackman CP, Muoio DM, Bursac N. Electrical stimulation increases hypertrophy and metabolic flux in tissue-engineered human skeletal muscle. Biomaterials. 2019;198:259–269. 30180985 10.1016/j.biomaterials.2018.08.058 PMC6395553

[69] Fujita H, Nedachi T, Kanzaki M. Accelerated de novo sarcomere assembly by electric pulse stimulation in C2C12 myotubes. Exp Cell Res. 2007;313(9):1853–1865. 17425954 10.1016/j.yexcr.2007.03.002

[70] Ahadian S, Ramón-Azcón J, Ostrovidov S, Camci-Unal G, Kaji H, Ino K, Shiku H, Khademhosseini A, Matsue T. A contactless electrical stimulator: Application to fabricate functional skeletal muscle tissue. Biomed Microdevices. 2013;15(1):109–115. 22965808 10.1007/s10544-012-9692-1

[71] Florini JR, Ewton DZ, Coolican SA. Growth hormone and the insulin-like growth factor system in myogenesis. Endocr Rev. 1996;17(5):481–517. 8897022 10.1210/edrv-17-5-481

[72] Ahmad SS, Ahmad K, Lee EJ, Lee Y-H, Choi I. Implications of insulin-like growth factor-1 in skeletal muscle and various diseases. Cells. 2020;9(8):1773. 32722232 10.3390/cells9081773 PMC7465464

[73] Guan X, Yan Q, Wang D, Du G, Zhou J. IGF-1 signaling regulates mitochondrial remodeling during myogenic differentiation. Nutrients. 2022;14(6):1249. 35334906 10.3390/nu14061249 PMC8954578

[74] Syverud BC, VanDusen KW, Larkin LM. Effects of dexamethasone on satellite cells and tissue engineered skeletal muscle units. Tissue Eng A. 2016;22(5–6):480–489. 10.1089/ten.tea.2015.0545 PMC4800275 26790477

[75] Stölting MNL, Arnold AS, Haralampieva D, Handschin C, Sulser T, Eberli D. Magnetic stimulation supports muscle and nerve regeneration after trauma in mice. Muscle Nerve. 2016;53(4):598–607. 26202157 10.1002/mus.24780 PMC5130145

[76] Yamamoto Y, Ito A, Jitsunobu H, Yamaguchi K, Kawabe Y, Mizumoto H, Kamihira M. Hollow fiber bioreactor perfusion culture system for magnetic force-based skeletal muscle tissue engineering. J Chem Eng Jpn. 2012;45(5):348–354.

[77] Pajčin I, Knežić T, Savic Azoulay I, Vlajkov V, Djisalov M, Janjušević L, Grahovac J, Gadjanski I. Bioengineering outlook on cultivated meat production. Micromachines. 2022;13(3):402. 35334693 10.3390/mi13030402 PMC8950996

[78] Kamei N, Adachi N, Ochi M. Magnetic cell delivery for the regeneration of musculoskeletal and neural tissues. Regen Ther. 2018;9:116–119. 30525082 10.1016/j.reth.2018.10.001 PMC6222975

[79] Nakabayashi A, Kamei N, Sunagawa T, Suzuki O, Ohkawa S, Kodama A, Kamei G, Ochi M. In vivo bioluminescence imaging of magnetically targeted bone marrow-derived mesenchymal stem cells in skeletal muscle injury model. J Orthop Res. 2013;31(5):754–759. 23192745 10.1002/jor.22282

[80] Liao I-C, Liu JB, Bursac N, Leong KW. Effect of electromechanical stimulation on the maturation of myotubes on aligned electrospun fibers. Cell Mol Bioeng. 2008;1(2–3):133–145. 19774099 10.1007/s12195-008-0021-y PMC2747747

[81] Langridge B, Griffin M, Butler PE. Regenerative medicine for skeletal muscle loss: A review of current tissue engineering approaches. J Mater Sci Mater Med. 2021;32(1):15. 33475855 10.1007/s10856-020-06476-5 PMC7819922

[82] Mantero S, Sadr N, Riboldi SA, Lorenzoni S, Montevecchi FM. A new electro-mechanical bioreactor for soft tissue engineering. J Appl Biomater Biomech. 2007;5(2):107–116. 20799180

[83] Lim D, Renteria ES, Sime DS, Ju YM, Kim JH, Criswell T, Shupe TD, Atala A, Marini FC, Gurcan MN, et al. Bioreactor design and validation for manufacturing strategies in tissue engineering. Bio-Des Manuf. 2022;5(1):43–63. 10.1007/s42242-021-00154-3 PMC8870603 35223131

[84] A study of in-vitro cell growth for bioreactor constructs. Discovery—the University of Dundee Research Portal. [accessed 28 Oct 2024] https://discovery.dundee.ac.uk/en/studentTheses/a-study-of-in-vitro-cell-growth-for-bioreactor-constructs

[85] Oragui E, Nannaparaju M, Khan WS. The role of bioreactors in tissue engineering for musculoskeletal applications. Open Orthop J. 2011;5(1):267–270. 21886691 10.2174/1874325001105010267 PMC3149843

[86] Yang A, Dong L. Engineering skeletal muscle tissue in bioreactor systems. Chin Med J. 2024;127(23):4130–4139. 25430462

[87] [Record Viewer] NLSP. [accessed 30 Jan 2025] https://nlsp.nasa.gov/view/lsdapub/lsda_hardware/IDP-LSDA_HARDWARE-0000000000000711

[88] Dvorak N, Liu Z, Mouthuy P-A. Soft bioreactor systems: A necessary step toward engineered MSK soft tissue? Front Robot AI. 2024;11:1287446. 38711813 10.3389/frobt.2024.1287446 PMC11070535

[89] Lee J, Kim H, Lim H-R, Kim YS, Hoang TTT, Choi J, Jeong G-J, Kim H, Herbert R, Soltis I, et al. Large-scale smart bioreactor with fully integrated wireless multivariate sensors and electronics for long-term in situ monitoring of stem cell culture. Sci Adv. 2024;10(7):eadk6714. 38354246 10.1126/sciadv.adk6714 PMC10866562

[90] DuRaine GD, Brown WE, Hu JC, Athanasiou KA. Emergence of scaffold-free approaches for tissue engineering musculoskeletal cartilages. Ann Biomed Eng. 2015;43(3):543–554. 25331099 10.1007/s10439-014-1161-y PMC4380596

[91] Collins CA, Olsen I, Zammit PS, Heslop L, Petrie A, Partridge TA, Morgan JE. Stem cell function, self-renewal, and behavioral heterogeneity of cells from the adult muscle satellite cell niche. Cell. 2005;122(2):289–301. 16051152 10.1016/j.cell.2005.05.010

[92] Mertens JP, Sugg KB, Lee JD, Larkin LM. Engineering muscle constructs for the creation of functional engineered musculoskeletal tissue. Regen Med. 2014;9(1):89–100. 24351009 10.2217/rme.13.81 PMC4482104

[93] Zhao Y, Wang EY, Lai FBL, Cheung K, Radisic M. Organs-on-a-chip: A union of tissue engineering and microfabrication. Trends Biotechnol. 2023;41(3):410–424. 36725464 10.1016/j.tibtech.2022.12.018 PMC9985977

[94] Agrawal G, Aung A, Varghese S. Skeletal muscle-on-a-chip: An in vitro model to evaluate tissue formation and injury. Lab Chip. 2017;17(20):3447–3461. 28871305 10.1039/c7lc00512a PMC6296378

[95] Wang Y, Yung P, Lu G, Liu Y, Ding C, Mao C, Li ZA, Tuan RS. Musculoskeletal organs-on-chips: An emerging platform for studying the nanotechnology–biology interface. Adv Mater. 2025;37(2):2401334. 38491868 10.1002/adma.202401334 PMC11733728

[96] Fuller H, Wei T-Y, Behrens M, Ruder W. The future application of organ-on-a-chip technologies as proving grounds for microbiorobots. Micromachines. 2020;11(10):947. 33092054 10.3390/mi11100947 PMC7589118

[97] Cook CA, Huri PY, Ginn BP, Gilbert-Honick J, Somers SM, Temple JP, Mao HQ, Grayson WL. Characterization of a novel bioreactor system for 3D cellular mechanobiology studies. Biotechnol Bioeng. 2016;113(8):1825–1837. 26825810 10.1002/bit.25946

[98] Bian W, Bursac N. Cellular/tissue engineering. IEEE Eng Med Biol Mag. 2008;27(5):109–113. 10.1109/MEMB.2008.928460 PMC2702132 18799400

[99] Wang Y, Liu M, Zhang W, Liu H, Jin F, Mao S, Han C, Wang X. Mechanical strategies to promote vascularization for tissue engineering and regenerative medicine. Burns Trauma. 2024;12:tkae039. 39350780 10.1093/burnst/tkae039 PMC11441985

[100] Namjoo AR, Abrbekoh FN, Saghati S, Amini H, Saadatlou MAE, Rahbarghazi R. Tissue engineering modalities in skeletal muscles: Focus on angiogenesis and immunomodulation properties. Stem Cell Res Ther. 2023;14(1):90. 37061717 10.1186/s13287-023-03310-x PMC10105969

[101] Samandari M, Quint J, Rodríguez-delaRosa A, Sinha I, Pourquié O, Tamayol A. Bioinks and bioprinting strategies for skeletal muscle tissue engineering. Adv Mater. 2022;34(12):2105883. 10.1002/adma.202105883 PMC8957559 34773667

[102] Cittadella Vigodarzere G, Mantero S. Skeletal muscle tissue engineering: Strategies for volumetric constructs. Front Physiol. 2014;5:362. 25295011 10.3389/fphys.2014.00362 PMC4170101

[103] Webber MJ, Khan OF, Sydlik SA, Tang BC, Langer R. A perspective on the clinical translation of scaffolds for tissue engineering. Ann Biomed Eng. 2015;43(3):641–656. 25201605 10.1007/s10439-014-1104-7 PMC4785597

[104] Tissue engineering market size and share report, 2030. [accessed 21 Feb 2025] https://www.grandviewresearch.com/industry-analysis/tissue-engineering-and-regeneration-industry

[105] TC-3. [accessed 18 Feb 2025]. https://ebersmedical.com/tissue-engineering/bioreactors/load-culture/tc-3

[106] BioTense. [accessed 18 Feb 2025] https://www.admet.com/wp-content/uploads/2015/07/ADMET-BioTense-Bioreactor-System-Brochure.pdf

[107] MCTX—Cellscale biomaterials testing. [accessed 18 Feb 2025] https://www.cellscale.com/products-cellscale-biomaterials-testing/mctx/

[108] Smith AF, Thanarak J, Pontin M, Green NH, Damian DD. Design and development of a robotic bioreactor for in vitro tissue engineering. Paper presented at: 2021 IEEE International Conference on Robotics and Automation (ICRA); 2021; Xi’an, China.

[109] Altman GH, Lu HH, Horan RL, Calabro T, Ryder D, Kaplan DL, Stark P, Martin I, Richmond JC, Vunjak-Novakovic G. Advanced bioreactor with controlled application of multi-dimensional strain for tissue engineering. J Biomech Eng. 2002;124(6):742–749. 12596643 10.1115/1.1519280

[110] Paek J, Song JW, Ban E, Morimitsu Y, Osuji CO, Shenoy VB, Huh DD. Soft robotic constrictor for in vitro modeling of dynamic tissue compression. Sci Rep. 2021;11(1):16478. 34389738 10.1038/s41598-021-94769-2 PMC8363742

[111] Asano Y, Okada K, Inaba M. Musculoskeletal design, control, and application of human mimetic humanoid Kenshiro. Bioinspir Biomim. 2019;14(3): Article 036011. 30708361 10.1088/1748-3190/ab03fc

[112] Jantsch M, Schmaler C, Wittmeier S, Dalamagkidis K, Knoll A. A scalable joint-space controller for musculoskeletal robots with spherical joints. Paper presented at: 2011 IEEE International Conference on Robotics and Biomimetics; 2011; Karon Beach, Thailand.

[113] Kharchenko A, Lippl J, Hostettler R. Embracing acceptance: Hugging robodies improves robot acceptance by the general population. Paper presented at: 2nd workshop Towards Robot Avatars, IEEE International Conference on Robotics and Automation (ICRA); 2023; London, UK.

[114] Mouthuy P-A, Snelling S, Hostettler R, Kharchenko A, Salmon S, Wainman A, Mimpen J, Paul C, Carr A. Humanoid robots to mechanically stress human cells grown in soft bioreactors. Commun Eng. 2022;1(1):2. 39075173 10.1038/s44172-022-00004-9 PMC10938861

[115] Feinberg AW. Biological soft robotics. Annu Rev Biomed Eng. 2015;17(1):243–265. 26643022 10.1146/annurev-bioeng-071114-040632

[116] Oveissi F, Fletcher DF, Dehghani F, Naficy S. Tough hydrogels for soft artificial muscles. Mater Des. 2021;203: Article 109609.

[117] Kurumaya S, Suzumori K, Nabae H, Wakimoto S. Musculoskeletal lower-limb robot driven by multifilament muscles. ROBOMECH J. 2016;3(1):18.

[118] Matsuda T, Kawakami R, Namba R, Nakajima T, Gong JP. Mechanoresponsive self-growing hydrogels inspired by muscle training. Science. 2019;363(6426):504–508. 30705187 10.1126/science.aau9533

[119] Elysium Robotics. [accessed 23 Oct 2024] https://www.elysium-robotics.com/about/

[120] Robodies—DEVANTHRO. [accessed 23 Oct 2024] https://www.devanthro.com/robodies/

[121] Sun Y, Duffy R, Lee A, Feinberg AW. Optimizing the structure and contractility of engineered skeletal muscle thin films. Acta Biomater. 2013;9(8):7885–7894. 23632372 10.1016/j.actbio.2013.04.036

[122] Anand SV, Ali MY, Saif MTA. Cell culture on microfabricated one-dimensional polymeric structures for bio-actuator and bio-bot applications. Lab Chip. 2015;15(8):1879–1888. 25712193 10.1039/c4lc01471e

[123] Wang Z, Klingner A, Magdanz V, Misra S, Khalil ISM. Soft bio-microrobots: Toward biomedical applications. Adv Intell Syst. 2024;6(2):2300093.

[124] Morimoto Y, Onoe H, Takeuchi S. Biohybrid robot powered by an antagonistic pair of skeletal muscle tissues. Sci Robot. 2018;3(18):eaat4440. 33141706 10.1126/scirobotics.aat4440

[125] Kinjo R, Morimoto Y, Jo B, Takeuchi S. Biohybrid bipedal robot powered by skeletal muscle tissue. Matter. 2024;7(3):948–962.

[126] Park S-J, Gazzola M, Park KS, Park S, di Santo V, Blevins EL, Lind JU, Campbell PH, Dauth S, Capulli AK, et al. Phototactic guidance of a tissue-engineered soft-robotic ray. Science. 2016;353(6295):158–162. 27387948 10.1126/science.aaf4292 PMC5526330

[127] Aydin O, Zhang X, Nuethong S, Pagan-Diaz GJ, Bashir R, Gazzola M, Saif MTA. Neuromuscular actuation of biohybrid motile bots. Proc Natl Acad Sci USA. 2019;116(40):19841–19847. 31527266 10.1073/pnas.1907051116 PMC6778261

[128] Guix M, Mestre R, Patiño T, De Corato M, Fuentes J, Zarpellon G, Sánchez S. Biohybrid soft robots with self-stimulating skeletons. Sci Robot. 2020;6(53):eabe7577. 10.1126/scirobotics.abe7577 34043566

[129] Ren X, Morimoto Y, Takeuchi S. Biohybrid hand actuated by multiple human muscle tissues. Sci Robot. 2025;10(99):eadr5512. 39937887 10.1126/scirobotics.adr5512

[130] Yang L, Zhang C, Wang R, Qin H, Zhang Y, Tan W, Yang J, Wang F, Liu L. Biosyncretic robots actuated by living materials. Adv Mater Technol. 2024;9(1):2301183.

[131] Liu L, Wu J, Chen B, Gao J, Li T, Ye Y, Tian H, Wang S, Wang F, Jiang J, et al. Magnetically actuated biohybrid microswimmers for precise photothermal muscle contraction. ACS Nano. 2022;16(4):6515–6526. 35290021 10.1021/acsnano.2c00833

[132] Raman R, Cvetkovic C, Uzel SGM, Platt RJ, Sengupta P, Kamm RD, Bashir R. Optogenetic skeletal muscle-powered adaptive biological machines. Proc Natl Acad Sci USA. 2016;113(13):3497–3502. 26976577 10.1073/pnas.1516139113 PMC4822586

[133] Sun L, Chen Z, Bian F, Zhao Y. Bioinspired soft robotic caterpillar with cardiomyocyte drivers. Adv Funct Mater. 2020;30(6):1907820.

[134] Dennis RG, Kosnik PE, Gilbert ME, Faulkner JA. Excitability and contractility of skeletal muscle engineered from primary cultures and cell lines. Am J Phys Cell Phys. 2001;280(2):C288–C295. 10.1152/ajpcell.2001.280.2.C288 11208523

[135] Almany L, Seliktar D. Biosynthetic hydrogel scaffolds made from fibrinogen and polyethylene glycol for 3D cell cultures. Biomaterials. 2005;26(15):2467–2477. 15585249 10.1016/j.biomaterials.2004.06.047

[136] Baryshyan AL, Domigan LJ, Hunt B, Trimmer BA, Kaplan DL. Self-assembled insect muscle bioactuators with long term function under a range of environmental conditions. RSC Adv. 2014;4(75):39962–39968. 25285210 10.1039/C4RA08438A PMC4180406

[137] Abmayr SM, Pavlath GK. Myoblast fusion: Lessons from flies and mice. Development. 2012;139(4):641–656. 22274696 10.1242/dev.068353 PMC3265056

[138] Pirosa A, Gottardi R, Alexander PG, Tuan RS. Engineering in-vitro stem cell-based vascularized bone models for drug screening and predictive toxicology. Stem Cell Res Ther. 2018;9(1):112. 29678192 10.1186/s13287-018-0847-8 PMC5910611

[139] Powell CA, Smiley BL, Mills J, Vandenburgh HH. Mechanical stimulation improves tissue-engineered human skeletal muscle. Am J Phys Cell Phys. 2002;283(5):C1557–C1565. 10.1152/ajpcell.00595.2001 12372817

[140] Heher P, Maleiner B, Prüller J, Teuschl AH, Kollmitzer J, Monforte X, Wolbank S, Redl H, Rünzler D, Fuchs C. A novel bioreactor for the generation of highly aligned 3D skeletal muscle-like constructs through orientation of fibrin via application of static strain. Acta Biomater. 2015;24:251–265. 26141153 10.1016/j.actbio.2015.06.033

[141] Quarta M, Cromie M, Chacon R, Blonigan J, Garcia V, Akimenko I, Hamer M, Paine P, Stok M, Shrager JB, et al. Bioengineered constructs combined with exercise enhance stem cell-mediated treatment of volumetric muscle loss. Nat Commun. 2017;8(1):15613. 28631758 10.1038/ncomms15613 PMC5481841

[142] Patel KH, Dunn AJ, Talovic M, Haas GJ, Marcinczyk M, Elmashhady H, Kalaf EG, Sell SA, Garg K. Aligned nanofibers of decellularized muscle ECM support myogenic activity in primary satellite cells in vitro. Biomed Mater. 2019;14(3): Article 035010. 30812025 10.1088/1748-605X/ab0b06

[143] Pei M, Hwangbo H, Kim G. Hierarchical fibrous collagen/poly(ε-caprolactone) structure fabricated with a 3D-printing process for tissue engineering applications. Compos Part B. 2023;259: Article 110730.

[144] Hruschka V, Saeed A, Slezak P, Cheikh al Ghanami R, Feichtinger GA, Alexander C, Redl H, Shakesheff K, Wolbank S. Evaluation of a thermoresponsive polycaprolactone scaffold for in vitro three-dimensional stem cell differentiation. Tissue Eng A. 2015;21(1–2):310–319. 10.1089/ten.TEA.2013.0710 25167885

[145] Aviss K, Gough J, Downes S. Aligned electrospun polymer fibres for skeletal muscle regeneration. Eur Cell Mater. 2010;19:193–204. 20467965 10.22203/ecm.v019a19

[146] Shin YC, Lee JH, Jin L, Kim MJ, Kim Y-J, Hyun JK, Jung T-G, Hong SW, Han D-W. Stimulated myoblast differentiation on graphene oxide-impregnated PLGA-collagen hybrid fibre matrices. J Nanobiotechnology. 2015;13(1):21. 25886153 10.1186/s12951-015-0081-9 PMC4379947

[147] Liu P, Sun L, Liu P, Yu W, Zhang Q, Zhang W, Ma J, Liu P, Shen J. Surface modification of porous PLGA scaffolds with plasma for preventing dimensional shrinkage and promoting scaffold–cell/tissue interactions. J Mater Chem B. 2018;6(46):7605–7613. 32254882 10.1039/c8tb02374c

[148] Gao H, Xiao J, Wei Y, Wang H, Wan H, Liu S. Regulation of myogenic differentiation by topologically microgrooved surfaces for skeletal muscle tissue engineering. ACS Omega. 2021;6(32):20931–20940. 34423201 10.1021/acsomega.1c02347 PMC8374903

[149] Fornetti E, de Paolis F, Fuoco C, Bernardini S, Giannitelli SM, Rainer A, Seliktar D, Magdinier F, Baldi J, Biagini R, et al. A novel extrusion-based 3D bioprinting system for skeletal muscle tissue engineering. Biofabrication. 2023;15(2): Article 025009. 10.1088/1758-5090/acb573 36689776

[150] Gong HY, Park J, Kim W, Kim J, Lee JY, Koh W-G. A novel conductive and micropatterned PEG-based hydrogel enabling the topographical and electrical stimulation of myoblasts. ACS Appl Mater Interfaces. 2019;11(51):47695–47706. 31794187 10.1021/acsami.9b16005

[151] Gilbert-Honick J, Ginn B, Zhang Y, Salehi S, Wagner KR, Mao HQ, Grayson WL. Adipose-derived stem/stromal cells on electrospun fibrin microfiber bundles enable moderate muscle reconstruction in a volumetric muscle loss model. Cell Transplant. 2018;27(11):1644–1656. 30298751 10.1177/0963689718805370 PMC6299198

[152] Fan T, Wang S, Jiang Z, Ji S, Cao W, Liu W, Ji Y, Li Y, Shyh-Chang N, Gu Q. Controllable assembly of skeletal muscle-like bundles through 3D bioprinting. Biofabrication. 2022;14(1): Article 015009. 10.1088/1758-5090/ac3aca 34788746

[153] Pollot BE, Rathbone CR, Wenke JC, Guda T. Natural polymeric hydrogel evaluation for skeletal muscle tissue engineering. J Biomed Mater Res. 2018;106(2):672–679. 10.1002/jbm.b.33859 28306190

[154] Takeda N, Tamura K, Mineguchi R, Ishikawa Y, Haraguchi Y, Shimizu T, Hara Y. In situ cross-linked electrospun fiber scaffold of collagen for fabricating cell-dense muscle tissue. J Artif Organs. 2016;19(2):141–148. 26472433 10.1007/s10047-015-0871-8

[155] Camman M, Joanne P, Brun J, Marcellan A, Dumont J, Agbulut O, Hélary C. Anisotropic dense collagen hydrogels with two ranges of porosity to mimic the skeletal muscle extracellular matrix. Biomater Adv. 2023;144: Article 213219. 36481519 10.1016/j.bioadv.2022.213219

[156] Rieu C, Parisi C, Mosser G, Haye B, Coradin T, Fernandes FM, Trichet L. Topotactic fibrillogenesis of freeze-cast microridged collagen scaffolds for 3D cell culture. ACS Appl Mater Interfaces. 2019;11(16):14672–14683. 30913387 10.1021/acsami.9b03219

[157] Basurto IM, Muhammad SA, Gardner GM, Christ GJ, Caliari SR. Controlling scaffold conductivity and pore size to direct myogenic cell alignment and differentiation. J Biomed Mater Res A. 2022;110(10):1681–1694. 35762455 10.1002/jbm.a.37418 PMC9540010

[158] Yeo M, Kim GH. Anisotropically aligned cell-laden nanofibrous bundle fabricated via cell electrospinning to regenerate skeletal muscle tissue. Small. 2018;14(48):1803491. 10.1002/smll.201803491 30311453

[159] Yi H, Forsythe S, He Y, Liu Q, Xiong G, Wei S, Li G, Atala A, Skardal A, Zhang Y. Tissue-specific extracellular matrix promotes myogenic differentiation of human muscle progenitor cells on gelatin and heparin conjugated alginate hydrogels. Acta Biomater. 2017;62:222–233. 28823716 10.1016/j.actbio.2017.08.022 PMC8151673

[160] Murugan P, Yap WS, Ezhilarasu H, Suntornnond R, le QB, Singh S, Seah JSH, Tan PL, Zhou W, Tan LP, et al. Decellularised plant scaffolds facilitate porcine skeletal muscle tissue engineering for cultivated meat biomanufacturing. npj Sci Food. 2024;8(1):25. 38702314 10.1038/s41538-024-00262-1 PMC11068908

[161] Hickey RJ, Modulevsky DJ, Cuerrier CM, Pelling AE. Customizing the shape and microenvironment biochemistry of biocompatible macroscopic plant-derived cellulose scaffolds. ACS Biomater Sci Eng. 2018;4(11):3726–3736. 33429594 10.1021/acsbiomaterials.8b00178

[162] Freytes DO. Development and characterization of a biohybrid scaffold for regenerative medicine applications [dissertation]. [Pittsburgh (PA)]: University of Pittsburgh; 2008.

[163] Wolf MT, Daly KA, Brennan-Pierce EP, Johnson SA, Carruthers CA, D’Amore A, Nagarkar SP, Velankar SS, Badylak SF. A hydrogel derived from decellularized dermal extracellular matrix. Biomaterials. 2012;33(29):7028–7038. 22789723 10.1016/j.biomaterials.2012.06.051 PMC3408574

[164] Smoak MM, Hogan KJ, Grande-Allen KJ, Mikos AG. Bioinspired electrospun dECM scaffolds guide cell growth and control the formation of myotubes. Sci Adv. 2021;7(20):eabg4123. 33990336 10.1126/sciadv.abg4123 PMC8121426

[165] Lee H, Ju YM, Kim I, Elsangeedy E, Lee JH, Yoo JJ, Atala A, Lee SJ. A novel decellularized skeletal muscle-derived ECM scaffolding system for in situ muscle regeneration. Methods. 2020;171:77–85. 31278981 10.1016/j.ymeth.2019.06.027

[166] Chromiak JA, Shansky J, Perrone C, Vandenburgh HH. Bioreactor perfusion system for the long-term maintenance of tissue-engineered skeletal muscle organoids. In Vitro Cell Dev Biol Anim. 1998;34(9):694–703. 9794221 10.1007/s11626-998-0065-2

[167] Bardouille C, Lehmann J, Heimann P, Jockusch H. Growth and differentiation of permanent and secondary mouse myogenic cell lines on microcarriers. Appl Microbiol Biotechnol. 2001;55(5):556–562. 11414320 10.1007/s002530100595

[168] Moon DG, Christ G, Stitzel JD, Atala A, Yoo JJ. Cyclic mechanical preconditioning improves engineered muscle contraction. Tissue Eng A. 2008;14(4):473–482. 10.1089/tea.2007.0104 18399787

[169] Flaibani M, Luni C, Sbalchiero E, Elvassore N. Flow cytometric cell cycle analysis of muscle precursor cells cultured within 3D scaffolds in a perfusion bioreactor. Biotechnol Prog. 2009;25(1):286–295. 19224607 10.1002/btpr.40

[170] Donnelly K, Khodabukus A, Philp A, Deldicque L, Dennis RG, Baar K. A novel bioreactor for stimulating skeletal muscle in vitro. Tissue Eng Part C Methods. 2010;16(4):711–718. 19807268 10.1089/ten.TEC.2009.0125

[171] Candiani G, Riboldi SA, Sadr N, Lorenzoni S, Neuenschwander P, Montevecchi FM, Mantero S. Cyclic mechanical stimulation favors myosin heavy chain accumulation in engineered skeletal muscle constructs. J Appl Biomater Biomech. 2010;8(2):68–75. 20740468

[172] Corona BT, Machingal MA, Criswell T, Vadhavkar M, Dannahower AC, Bergman C, Zhao W, Christ GJ. Further development of a tissue engineered muscle repair construct in vitro for enhanced functional recovery following implantation in vivo in a murine model of volumetric muscle loss injury. Tissue Eng A. 2012;18(11-12):1213–1228. 10.1089/ten.tea.2011.0614 PMC3360508 22439962

[173] Cerino G, Gaudiello E, Grussenmeyer T, Melly L, Massai D, Banfi A, Martin I, Eckstein F, Grapow M, Marsano A. Three dimensional multi-cellular muscle-like tissue engineering in perfusion-based bioreactors. Biotechnol Bioeng. 2016;113(1):226–236. 26126766 10.1002/bit.25688

[174] Rimington RP, Capel AJ, Chaplin KF, Fleming JW, Bandulasena HCH, Bibb RJ, Christie SDR, Lewis MP. Differentiation of bioengineered skeletal muscle within a 3D printed perfusion bioreactor reduces atrophic and inflammatory gene expression. ACS Biomater Sci Eng. 2019;5(10):5525–5538. 33464072 10.1021/acsbiomaterials.9b00975

[175] Todros S, Spadoni S, Maghin E, Piccoli M, Pavan PG. A novel bioreactor for the mechanical stimulation of clinically relevant scaffolds for muscle tissue engineering purposes. Processes. 2021;9(3):474.

[176] Rojas-Rojas L, Espinoza-Álvarez ML, Castro-Piedra S, Ulloa-Fernández A, Vargas-Segura W, Guillén-Girón T. Muscle-like scaffolds for biomechanical stimulation in a custom-built bioreactor. Polymers. 2022;14(24):5427. 36559794 10.3390/polym14245427 PMC9781371

[177] Kamal KY, Othman MA, Kim J-H, Lawler JM. Bioreactor development for skeletal muscle hypertrophy and atrophy by manipulating uniaxial cyclic strain: Proof of concept. npj Microgravity. 2024;10(1):62. 38862543 10.1038/s41526-023-00320-0 PMC11167039

